# Reductive Evolution and Diversification of C5-Uracil Methylation in the Nucleic Acids of Mollicutes

**DOI:** 10.3390/biom10040587

**Published:** 2020-04-10

**Authors:** Pascal Sirand-Pugnet, Damien Brégeon, Laure Béven, Catherine Goyenvalle, Alain Blanchard, Simon Rose, Henri Grosjean, Stephen Douthwaite, Djemel Hamdane, Valérie de Crécy-Lagard

**Affiliations:** 1 INRAE, UMR BFP, University Bordeaux, 33882 Bordeaux Villenave D’Ornon, France; laure.beven@inrae.fr (L.B.); alain.blanchard@inrae.fr (A.B.); 2 IBPS, Biology of Aging and Adaptation, Sorbonne University, 7 quai Saint Bernard, CEDEX 05, F-75252 Paris, France; damien.bregeon@sorbonne-universite.fr (D.B.); catherine.goyenvalle@upmc.fr (C.G.); 3 Department of Biochemistry and Molecular Biology, University of Southern Denmark, Campusvej 55, DK-5230 Odense M, Denmark; simonro@bmb.sdu.dk (S.R.); srd@bmb.sdu.dk (S.D.); 4 Institute for Integrative Biology of the Cell (I2BC), French Atomic Energy and Energy Commission Alternatives, CNRS, Paris-Sud University, Paris-Saclay University, Gif-sur-Yvette CEDEX, 91198 Paris, France; henri4g@me.com; 5 Laboratory of Biological Process Chemistry, CNRS-UMR 8229, College De France, Sorbonne University, 11 Place Marcelin Berthelot, CEDEX 05, 75231 Paris, France; 6 Department of Microbiology and Cell Science, University of Florida, Gainesville, FL 32611, USA; 7 Genetics Institute, University of Florida, Gainesville, FL 32610, USA

**Keywords:** base modification, methyltransferases, flavoenzymes, tRNA, rRNA, mycoplasmas, spiroplasmas, acholeplasmas, evolution, minimal cell, moonlighting function

## Abstract

The C5-methylation of uracil to form 5-methyluracil (m^5^U) is a ubiquitous base modification of nucleic acids. Four enzyme families have converged to catalyze this methylation using different chemical solutions. Here, we investigate the evolution of 5-methyluracil synthase families in *Mollicutes*, a class of bacteria that has undergone extensive genome erosion. Many mollicutes have lost some of the m^5^U methyltransferases present in their common ancestor. Cases of duplication and subsequent shift of function are also described. For example, most members of the Spiroplasma subgroup use the ancestral tetrahydrofolate-dependent TrmFO enzyme to catalyze the formation of m^5^U54 in tRNA, while a TrmFO paralog (termed RlmFO) is responsible for m^5^U1939 formation in 23S rRNA. RlmFO has replaced the S-adenosyl-L-methionine (SAM)-enzyme RlmD that adds the same modification in the ancestor and which is still present in mollicutes from the Hominis subgroup. Another paralog of this family, the TrmFO-like protein, has a yet unidentified function that differs from the TrmFO and RlmFO homologs. Despite having evolved towards minimal genomes, the mollicutes possess a repertoire of m^5^U-modifying enzymes that is highly dynamic and has undergone horizontal transfer.

## 1. Introduction

Methylation reactions are essential for a large number of cellular processes including DNA synthesis, genome protection against restriction systems, gene expression and regulation, and post-transcriptional modification of RNAs [1,2]. Among these reactions, the C5-methylation of uracil yielding 5-methyluracil (m^5^U) constitutes one of the most common nucleic acids modifications found both in DNA and RNA. 5-Methyluracil is ubiquitous in DNA as one of the four canonical bases, deoxythymidine (dT or deoxy m^5^U), and is formed by the de novo methylation of the deoxyuridine monophosphate (dUMP) precursor to deoxythymidine monophosphate (dTMP) [3]. In RNA, 5-methyluracil is present as ribothymidine (rT or m^5^U) and is synthetized post-transcriptionally [1,2]. So far, m^5^U has been found at position 54 in the T-loop of tRNAs (m^5^U54), where it is conserved in most organisms, and at the corresponding position in bacterial tmRNA (formerly 10Sa RNA) [4,5,6]. However, in some organisms, including a few mycoplasmas, m^5^U54 is absent from the tRNA molecules [7,8,9]. This modification also occurs in some bacterial 23S rRNAs at positions m^5^U747 and m^5^U1939 [10,11]. The presence of m^5^U stabilizes RNA molecules by increasing the intrinsic stacking power and rigidity of the loop containing the modified nucleosides [12,13,14], also allowing protection against degradation by specific nucleases [15,16]. Furthermore, 5-methyluracil has recently been shown to be a component of the polyoxin antibiotic (PolB) produced by *Streptomyces cacaoi* subsp. *asoensis*, where antibiotic synthesis is initiated by C5-methylation of UMP to m^5^UMP [17]. This finding indicates that 5-methyluracil can engage in additional pathways other than those specifically linked to DNA and RNA metabolism.

**Figure 1:**
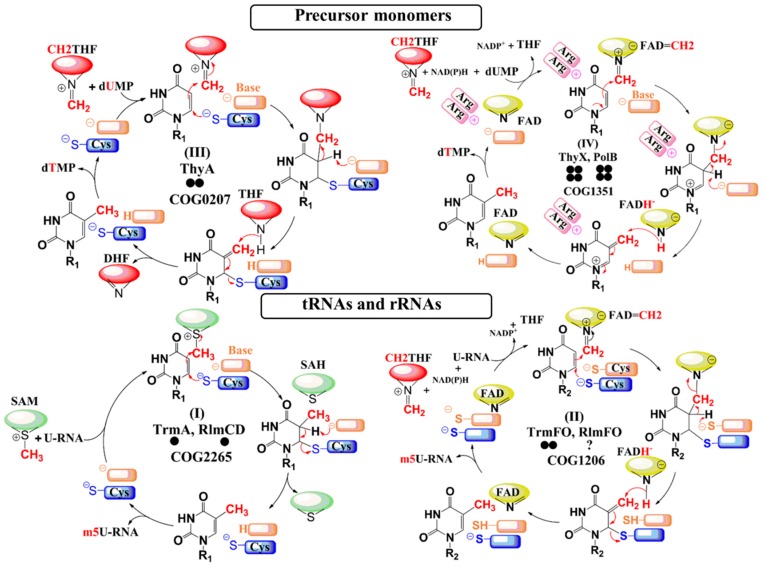
Enzymatic mechanisms of the *C5*-uracil methyltransferases. The cofactors and flavin coenzyme derivatives are represented as simplified pictograms. The pteridin moiety of the folate derivatives is colored in red, whereas the S-adenosylhomocysteine and the isoalloxazine ring of flavin adenine dinucleotide (FAD) are colored in green and yellow, respectively. Refer to Figure S1 for the chemical structures of the cofactors and FAD coenzyme. For more details about the chemical mechanisms of these enzymatic systems, see references [18,19,20,21,22,25,26,39,40]. Residues playing the role of base in the catalytic cycle are in orange, whereas the nucleophiles are in blue. The bold circles below each protein’s name represent the oligomeric state of the enzyme.

Interestingly, methylation of C5-uracil can occur via four distinct mechanisms involving four structurally unrelated enzyme families with distinct evolutionary origins, highlighting how independent solutions have evolved to catalyze the same reaction [3,18,19,20,21] (Table 1). The most widespread enzyme involved in dTMP synthesis is the homodimeric thymidylate synthase ThyA (COG0207), encoded in most prokaryotes by the *thyA* gene and by its ortholog in humans [22,23]. This enzyme uses *N5,N10*-methylenetetrahydrofolate (CH_2_THF) as a methylene donor and THF as a reducing agent (Table 1). In the reaction pathway, a conserved cysteine in the enzyme activates the C6-position of dUMP via a classical Michael addition, increasing the nucleophilic character of the C5-position. Direct transfer of CH_2_ from CH_2_THF then takes place via the formation of a transient enzyme–dUMP–folate covalent intermediate. Removal of the *H5*-proton promotes the heterolytic cleavage of the C-C bond between CH_2_THF and dUMP, yielding an exocyclic methylene, which is ultimately reduced to a methyl group by THF (Figure 1).

The subsequent discovery of the thymidylate synthase ThyX (a distinct COG1351 of ThyA), established the existence of an alternate mechanism for dUMP methylation [24]. ThyX is less widespread than its ThyA counterpart and mainly found in prokaryotes and a few eukaryotes. ThyX is a homotetrameric flavoenzyme that employs the flavin adenine dinucleotide (FAD) as a coenzyme, with nicotinamide adenine dinucleotide phosphate (NADPH) as the initial source of hydride and CH_2_THF as a methylene donor (Table 1). In contrast to ThyA, the CH_2_ moiety from CH_2_THF is first transferred to the *N5*-atom of reduced flavin (FADH^-^), obtained from the preliminary reaction of FAD and NADPH, leading to the previously unseen flavin iminium species FAD=CH_2_, which acts as the bona fide dUMP methylating agent [25,26] (Figure 1). An additional mechanistic feature that distinguishes ThyA from ThyX is that the active site of ThyX has two essential conserved arginine residues that become polarized to activate dUMP [27]. Following the activation step, the electrophilic methylene on FAD=CH_2_ is passed to the *C5*-dUMP via the formation of a transient dUMP-CH_2_-FAD adduct, which eventually breaks down after abstraction of the *H5* proton. The resulting exocyclic methylene is then reduced by a hydride donated by FADH^-^, as opposed to the case of ThyA where reduction occurs via THF. This flavin- and folate-dependent mechanism is shared by PolB, a ThyX paralog also belonging to COG1351 that is involved in *C5*-UMP methylation during polyoxin biosynthesis [17]. PolB can also methylate dUMP but with a lower catalytic efficacy than its natural substrate (UMP).

Formation of m^5^U in tRNAs can also be catalyzed by two fundamentally different pathways, one of which is S-adenosyl-L-methionine (SAM)-dependent while the other uses flavin and folate [28,29,30]. The SAM-dependent pathway is the most common and uses a mechanism similar to 5-methylcytosine DNA and RNA methyltransferases. In most characterized organisms, m^5^U54 in tRNAs and at the corresponding position in bacterial tmRNA is synthesized by a SAM-dependent methyltransferase, such as the *Escherichia coli* enzyme TrmA [31,32,33]. In addition, m^5^U1939 (present in most bacterial 23S rRNAs) and m^5^U747 (less common and found mainly in Gram-negative beta-, epsilon-, and gammaproteobacteria) are catalyzed by the SAM-dependent RlmD (formerly RumA) and RlmC (formerly RumB) methyltransferases, respectively [34,35]. Interestingly, both of these rRNA m^5^U modifications in *Bacillus subtilis* are catalyzed by the same SAM-dependent RNA methyltransferase RlmCD, showing that dual target specificity is possible [36]. All these m^5^U tRNA or rRNA methyltransferases are members of the same superfamily (COG2265) and thus share a common ancestry. Furthermore, all use a simple mechanism based on direct transfer of the methyl group from the electrophilic carbon of the SAM cofactor to the activated *C5*-uracil. As in ThyA, this carbon is activated by a conserved cysteine that plays the role of nucleophile (Table 1 and Figure 1). Another COG2265 family member, YfjO, is encoded in the *B. subtilis* genome and, although its function remains unknown, this putative enzyme has been included in our screening process.

Alternative mechanisms of RNA m^5^U modification, which are analogous to dTMP synthesis, are seen with the two flavoenzyme homologs, namely TrmFO that adds the m^5^U54 modification in some tRNAs [30] and RlmFO that is responsible for the m^5^U1939 modification in 23S rRNA [37]. These RNA methyltransferases belong to a distinct COG1206. To date, only one case of m^5^U1939 formation by RlmFO has been described, and this is in the mollicutes *Mycoplasma capricolum* subsp. *capricolum* [37]. TrmFO, while apparently more common and found mainly in Gram-positive bacteria and some mollicutes [30,38], remains much less prevalent than the SAM-dependent pathway. Although the TrmFO/RlmFO enzymes employ a chemical mechanism using FAD=CH_2_ as the methylating agent, similar to that of ThyX [18,39,40], they differ from ThyX in their means of substrate activation where TrmFO/RlmFO rely on a conserved cysteine nucleophile in a manner similar to ThyA [41] (Table 1 and Figure 1).

These observations show that while fundamentally different types of m^5^U-modifying enzyme have evolved convergently to modify the same nucleotide target, other m^5^U-modifying enzymes that are structurally similar have diverged in their functions to modify different RNA sites. In the present study, we investigated how such phenomena might have evolved within the mollicutes. These organisms, with their small genome sizes of ~1 Mbp on average, represent excellent models for defining a minimal set of genes required for life and, more generally, for studying the mechanisms of genome reduction and evolution [42,43,44]. We studied a diverse array of mollicutes to systematically explore the distribution and function of two different SAM- and folate-dependent families of RNA m^5^U methyltransferases (COG1206 and COG2265), and mapped the complex patterns of acquisition and loss of the genes for these enzymes. The methylation targets of the enzymes were established. Furthermore, we note that the experimental data did not always coincide with bioinformatics predictions, which emphasizes the necessity of empirical testing to obtain reliable functional annotations of these enzymes.

## 2. Materials and Methods

### 2.1. In silico Genome and Protein Analyses

*Escherichia coli* and *Bacillus subtilis* m^5^U modification enzymes were used in blastp searches for mollicutes homologs in the MolliGen (http://molligen.org) database [45]. MolliGen and MBGD (http://mbgd.genome.ad.jp/) [46] databases were used to study the genomic contexts of genes of interest. The phylogenetic tree of mollicutes was generated using the maximum likelihood method from the concatenated multiple sequence alignments of 79 selected orthologous proteins involved in translation [47]. For phylogenetic analyses of TrmFO and RlmD homologs, protein alignments were obtained with MUSCLE (https://www.ebi.ac.uk/Tools/msa/muscle/) and cured from unreliable positions using Gblocks [48]. Phylogenetic trees were then inferred using the maximum likelihood method using the PhyML software implemented at phylogeny.fr (http://www.phylogeny.fr) [49]. An overview of conserved positions was obtained from protein alignments created using Jalview [50].

### 2.2. Functional Domain Analysis and Secondary Structure Prediction

The TrmFO homologs were modelled using the SWISS-MODEL server (https://swissmodel.expasy.org) [51]. The (Quaternary Structure Quality Estimate) QSQE score is a number between 0 and 1, reflecting the expected accuracy of the interchain contacts for a model built based a given alignment and template. In general, a higher QSQE is “better”, while this complements the (Global Model Quality Estimation) GMQE score that estimates the accuracy of the tertiary structure of the resulting model. QSQE is only computed for the top-ranked templates. Protein electrostatic surfaces were calculated using APBS (v1.4) software [52]. Calculations were performed at 310 K with 150 mM NaCl with the same grid size (193,193,161) in all cases showing electrostatic potential within ± 3 kTe-1. 

### 2.3. RNA Extraction and HPLC Analysis of tRNAs

Mollicutes cells were grown to late log phase and harvested by centrifugation at 10,000× *g* for 20 min. Cells (0.5× *g*) were washed twice by resuspending in 100 mL buffer A (50 mM Tris-Cl pH 7.2, 10 mM MgCl_2_, 100 mM NH_4_Cl) and pelleting by centrifugation. Cells were lysed by sonication at 4 °C in 10 mL buffer A. Cell debris containing the chromosomal DNA was removed by centrifugation at 15,000× *g* for 10 min. The supernatant was extracted with phenol/chloroform and total RNA was recovered by ethanol precipitation before redissolving in 100 μL H_2_O. Half of each sample was kept for rRNA analysis (below), and the remainder was passed through a Nucleobond^®^ RNA/DNA 400 column (Macherey-Nagel, Düren, Germany) to isolate the tRNA fraction. Bulk tRNAs were digested to completion to form nucleosides [53] before being subjected to reverse-phase chromatography on an Agilent Technologies 1200 series HPLC (Santa Clara, CA, USA) with a Phenomenex Luna C18 column (Torrance, CA, USA) (2 × 250 mm, 5 μm particles, 100 Å pores). Nucleosides were eluted as described previously [37,53] with 40 mM ammonium acetate pH 6 and a linear gradient of 0% to 40% acetonitrile, detecting eluents at 260 nm.

### 2.4. Analysis of RNA by Matrix-Assisted Laser Desorption/Ionization Mass Spectrometry (MALDI-MS) 

Total RNA extracts from mollicutes cells were analyzed within the 23S rRNA regions previously shown in other organisms to contain m^5^U methylations. In each case, 100 pmol of total RNA were hybridized to 500 pmol of the 48-mer deoxyoligonucleotide, 5′-GCCACAAGTCATCCAAAGTCTTTTCAACGAATACTGGTTCGGTCCTCC, complementary to the sequence G725-C772 in domain II of 23S rRNA, or to the 55-mer 5′-CGGGTCAGAATTTACCTGACAAGGAATTTCGCTACCTTAGGACCGTTATAGTTAC, complementary to the sequence G1910-G1964 within domain IV of 23S rRNA. The exposed regions within the RNAs were digested away with nucleases, and the sequences protected by hybridization were separated by gel electrophoresis [54,55]. The protected rRNA fragments were extracted and digested with RNases A or T1 in aqueous solution and analyzed by MALDI-MS (Ultraflextreme, Bruker Daltonics, Hamburg, Germany). Spectra were recorded in reflector and positive-ion mode and processed using Flexanalysis (Bruker Daltonics) [56].

### 2.5. Complementation Tests of ∆thyA::kan E. coli Strain

A transition mutation of A to G was introduced at the 5’-end of MCAP_0613 to create an NcoI site, and the gene was cloned into the NcoI and PstI restriction sites of pBAD24. This change corresponds to a K2E substitution at the *N-*terminus of the MCAP_0613 protein. *E. coli* strains BW25113 (F- ∆(*araD-araB*)567, *lacZ*4787(∆)::*rrnB*-3, LAM-*rph*-1, DE(*rhaD-rhaB*)568, *hsd*R514) and its ∆*thyA::kan* derivative were transformed with pBAD24 and pBAD24::MCAP_0613. Cells were grown to OD600 of 0.8 in LB medium supplemented with ampicillin (100 µg/mL^−1^) and thymidine (0.3 mM). Cells were washed twice in water and 10 µL of serial dilutions were spotted onto LB plates containing ampicillin and arabinose (0.02%) and, in some cases, supplemented with thymidine at 0.3 mM. Cells were incubated at 37 °C for 24 h. Growth rates and yields were measured in liquid cultures following standard procedures.

## 3. Results

### 3.1. Distribution of Predicted 5-Methyluracil Synthesis Enzymes in Mollicutes

Thirty-nine representatives of the main phylogenetic subgroups of the class *Mollicutes* were selected as a reference set for this study (Figure S2 and Table S1). In order to predict the repertoire of enzymes involved in dUMP, rRNA, and tRNA 5-methyluracil modifications in mollicutes, blastp queries against deduced proteomes of the selected reference set were conducted using the following input sequences: ThyA (b2827); TrmA (b3965); RlmC (b0859), and RlmD (b2785) from *E. coli*; TrmFO (BSU16130), RlmCD, and YfjO (BSU08020) from *B. subtilis*; ThyX (P9WG57) from *Mycobacterium tuberculosis*; and PolB (Uniprot: C1IC19) from *S. cacaoi* subsp. *asoensis.* We also searched for the presence of homologs of the deoxyT salvage enzyme thymidine kinase Tdk (b1238, BSU37060) because, in the absence of ThyA, Tdk becomes essential to provide dTMP precursors. If no candidate enzymes were found using this first approach, further analyses were performed using mollicutes homologs as queries and tblastn. Examples of paralogy were detected with more than one copy of TrmFO or YfjO homologs per genome, and in these instances, further phylogeny and synteny analyses were carried out to separate the subfamilies. 

TrmFO homologs were found in twelve of the thirty-nine mollicutes species, with ten of the genomes containing more than one copy (Table S2). In order to clarify the evolutionary relationships between TrmFO-related homologs, a phylogenetic tree was constructed adding other TrmFO homologs identified in Gram-positive bacteria and in recently sequenced mollicutes genomes (Figure S3). Two main groups supported by 100% statistical values were clearly identified. One of them includes the TrmFO-related homolog (MCAP_0476) from *M. capricolum* subsp. *capricolum* that was previously shown to catalyze the formation of m^5^U1939 in 23S rRNA, and subsequently renamed RlmFO [37]. The genomic context region around RlmFO encodes genes that are highly conserved, even among remote species of the Spiroplasma phylogenetic group (Figure S4). The other well-defined group of TrmFO-related homologs includes the *M. capricolum* subsp. *capricolum* paralog MCAP_0613, the function of which remains unknown, and we have renamed this subgroup “TrmFO-like”. Analysis of the genomic context of TrmFO-like homologs showed only a moderate conservation between related mycoplasmas from the Mycoides cluster (Figure S5). The remaining TrmFO-related homologs were distributed between two other subgroups supported by statistical values of 85% and 87%, separating proteins in the Acholeplasma clade from the rest of the mollicutes. Gene synteny was however observed among genomic regions surrounding all other *trmFO*-related homologs (Figure S6), showing some conservation of gene order with *trmFO* homologs from other Gram-positive bacteria including *B. subtilis*. This conserved synteny suggests that all these *trmFO*-related genes are true orthologs of the genuine ancestral *trmFO* present in the common ancestor of Gram-positive bacteria and mollicutes, and we now refer to them as the TrmFO subgroup.

**Figure 2:**
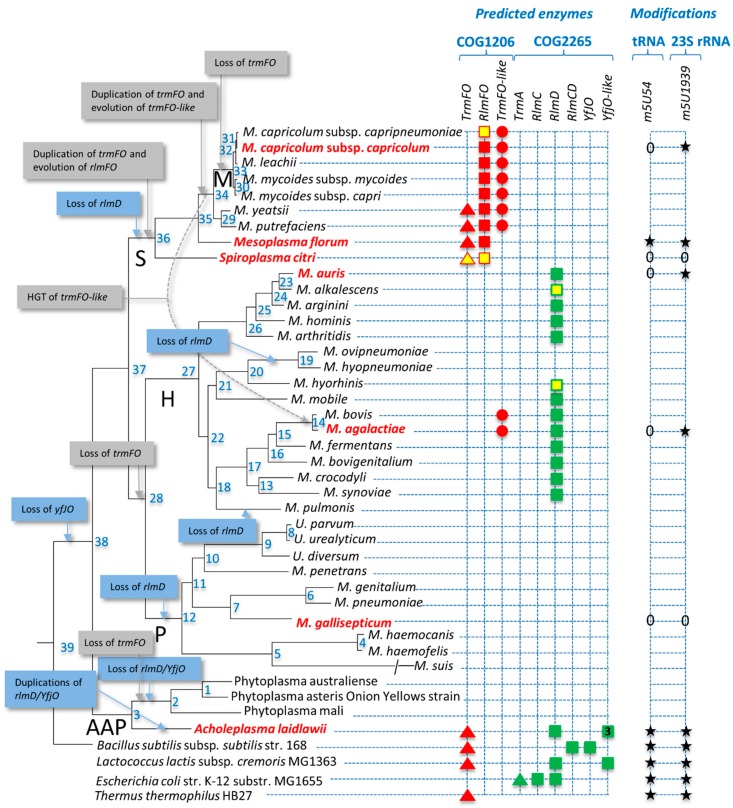
Occurrence of genes involved in rRNA/tRNA modification in mollicutes. The phylogenetic tree was inferred using the maximum likelihood method from the concatenated multiple alignments of 79 proteins encoded by genes present at one copy in each genome. Nodes are numbered. Main phylogenetic groups are indicated as follows: S, Spiroplasma; H, Hominis; P, Pneumoniae; AAP, Acholeplasma/Phytoplasma; M, Mycoides cluster of ruminant mycoplasmas. Non-cultivated species are framed by a red dotted rectangle. Species analyzed by MS are indicated in bold red. Targets are indicated as follows: square, rRNA; triangle, tRNA; circle, unknown. Symbols are colored as follows: red, folate-dependent enzymes; green, SAM-dependent enzymes; yellow, predicted pseudogene. Predicted evolution events are indicated in grey boxes. nd, not determined.

Four RlmD homologs were identified in *Acholeplasma laidlawii*, an unexpectedly high number for such a genome-reduced bacterium (1.5 Mpb). Further investigation showed that most Acholeplasma species also have four homologs, and this seems to be a recent expansion in the group (Figure S7, Table S3). The full comparative genomic analysis with accession numbers of all identified proteins, and the grouping in the different paralogous subgroups (Table S2), is summarized in Figure 2. The functional hypotheses derived from this information are presented below.

### 3.2. Synthesis by ThyA Homologs and deoxyT Salvage are the Two Main Routes to dTMP Synthesis in Mollicutes

The phylogenetic distribution analysis of enzyme involved in dTMP synthesis revealed that there are no homologs of ThyX and PolB in mollicutes. In addition, ~40% (16/39) of the mollicutes genomes analyzed encode a ThyA homolog, and these are scattered over different phylogenetic subgroups (Table S2). Furthermore, all but one of the mollicutes (*Mycoplasma bovigenitalium* cl-51080) encode Tdk homologs (Table S2) and, as no other specific enzyme for dTMP synthesis is presently known, this would suggest that thymidine salvage is the major pathway for this process in mollicutes. 

### 3.3. Most Mollicutes Have Lost the m^5^U54 Modification in tRNA

**Figure 3:**
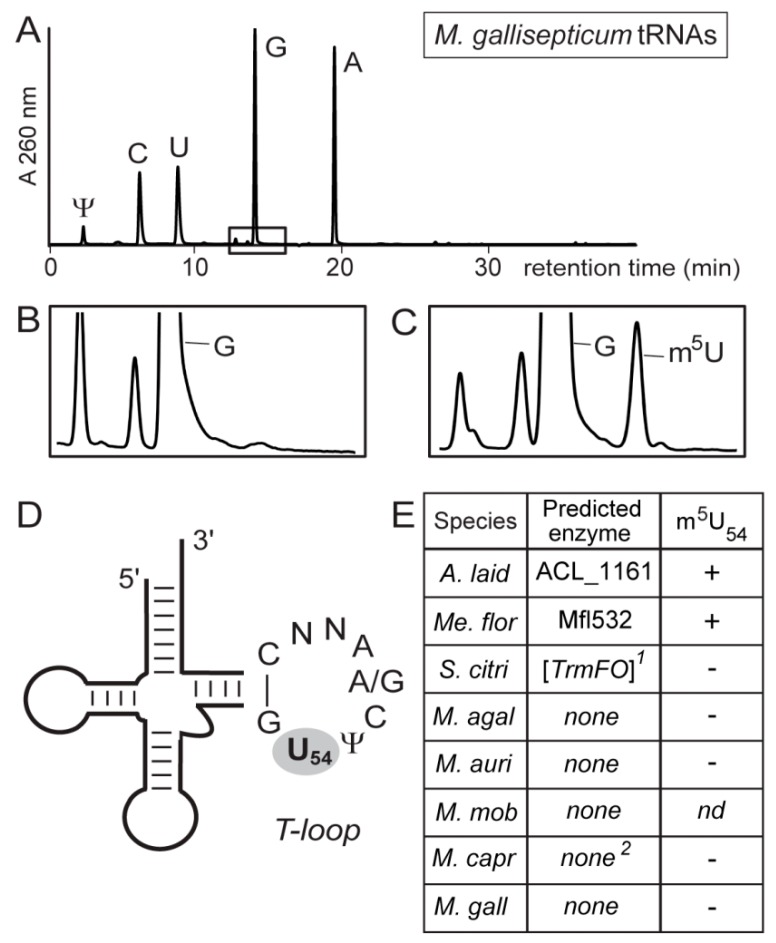
HPLC profiles of tRNA nucleosides from mollicutes bulk tRNAs. (**A**) The digestion products of total tRNAs from *M. gallisepticum* showing the positions of the unmodified nucleosides and pseudouridine detected by their absorbance at 260 nm (A260 nm). Under the conditions used here, m^5^U elutes in the boxed region after guanosine at approximately 15 min. Enlargement of the boxed region (**B**) for *M. gallisepticum* and (**C**) for *Me. florum,* where only the tRNAs of this latter strain contain m^5^U. (**D**) Schematic of the mollicutes tRNAs showing the consensus sequence of the T-loop nucleotides and the position of U54. (**E**) Mollicutes species shown empirically to have (+) or not have (-) m^5^U54 in their tRNAs. This modification correlates with the presence of an active TrmFO enzyme predicted by bioinformatics. Species abbreviations: *A. laid*, *A. laidlawii*; *Me. flor*, *Me. florum*; *S. citri*, *S. citri*; *M. agal*, *M. agalactiae*; and the additional *Mycoplasma* species *M. auris* (*M. auri*), *M. mobile* (*M. mob*), *M. capricolum* subsp. *capricolum* (*M. capr*), and *M. gallisepticum* (*M. gall*). ^1^ The *trmFO* sequence in *S. citri* is a nonfunctional pseudogene; ^2^ *M. capr* has no *trmFO*, but possesses a functional *rlmFO*. nd, not determined.

No bacterial TrmA homolog was found in any of the mollicutes (Table S2) and the majority (34/39) also lack a TrmFO homolog, suggesting that the corresponding tRNA modification has been lost in most members of the class. Putative orthologs of TrmFO are present only in *A. laidlawii* and in four members of the Spiroplasma group, where in one of these, *Spiroplasma citri*, it is a pseudogene (see below) and thus presumably in the process of being lost (Figure 2). In order to test our functional predictions, we analyzed the tRNAs from seven strains for the presence of m^5^U. A combination of strains was chosen to cover the various permutations of absence and presence of TrmFO, RlmFO, and TrmFO-like homologs. The tRNA m^5^U modification was found to be present only when a strain encodes what appears to be a functional *trmFO* gene (Figure 3).

This modification is absent in the tRNAs from *M. capricolum* subsp. *capricolum,* which encodes both RlmFO and TrmFO-like genes but no TrmFO, and is also absent in *S. citri* which encodes a TrmFO pseudogene.

### 3.4. Mollicutes Modify 23S rRNA m^5^U1939 via either RlmD or RlmFO

**Figure 4:**
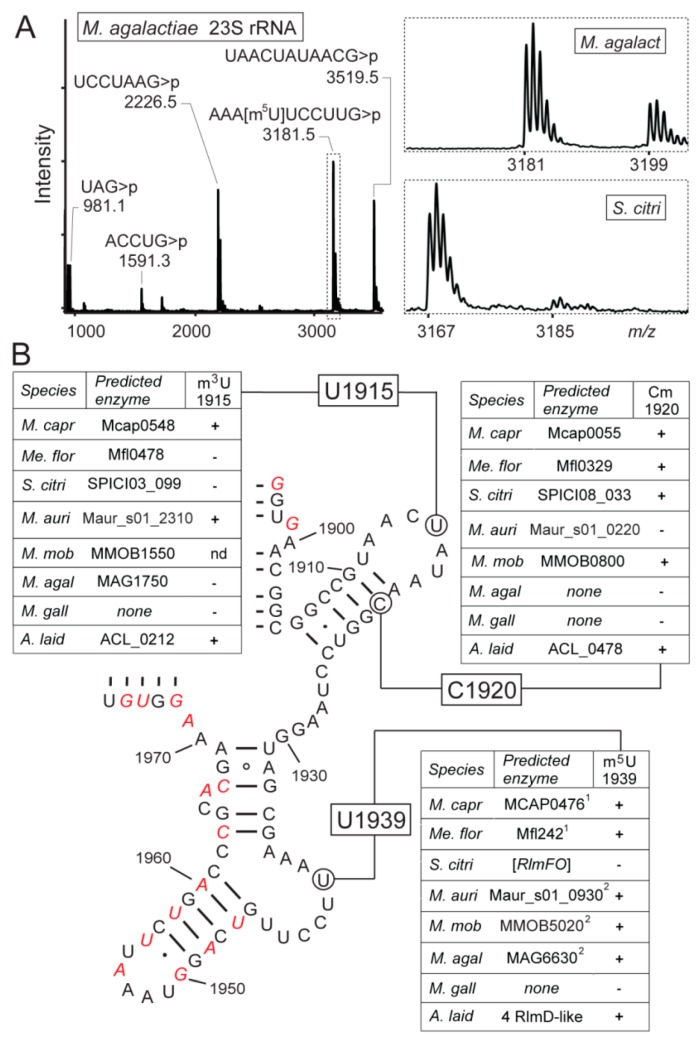
(**A**) MALDI-MS spectrum of RNase T1 fragments from the *M. agalactiae* 23S rRNA region around nucleotide U1939. The fragment sequences are shown above the peaks with their protonated masses (*m/z*); the *m/z* measurements are for fragments with a cyclic 3´-phosphate (>p), and are within 0.1 Da of the theoretical monoisotopic values. The spectral region (boxed) with nucleotide 1936-1945 fragment is expanded, showing that the *M. agalactiae* sequence at *m/z* 3181 contains the m^5^U1939 modification. The lower box shows the same spectral region from *S. citri* 23S rRNA, where the corresponding fragment at *m/z* 3167 is unmethylated. Minor peaks of the hydrated linear fragments with the same sequence (+ 18 Da) are visible in both spectra. Also of note here is the peak corresponding to *M. agalactiae* nucleotides 1911-1921, which has an m/z value of 3519 showing that U1915 and C1920 are both unmodified. These two nucleotides are analyzed in greater detail in Figure S8. (**B**) Schematic of the mollicutes 23S rRNA secondary structure around the potential methylation sites at nucleotides U1915, C1920, and U1939. The structure shown here is highly conserved in all bacteria and varies among the mollicutes only at the nucleotides highlighted in red (where the sequence specific for *M. capricolum* subsp. *capricolum* is shown here). The tables list the bioinformatics predictions for the presence of methyltransferases in *A. laidlawii*, *Me. florum*, *S. citri*, *M. agalactiae*; *M. auris*, *M. mobile*, *M. capricolum* subsp. *capricolum*, and *M. gallisepticum* (abbreviated as in Figure 3); the presence of the rRNA modifications was ascertained empirically, as above. Where present, the m^5^U1939 modification was added either by an RlmD homolog or an RlmFO homolog; *S. citri* appears to contain a nonfunctional pseudo-RlmFO. *A. laidlawii* possesses four RlmD paralogs, and it is not yet clear which of these has the m^5^U1939 modification function. nd, not determined.

Some species of mollicutes possess a homolog of either RlmD or RlmFO that directs the 23S rRNA modification at m^5^U1939 (Figure 4). The genes encoding these two enzymes appear mutually exclusive in these bacteria. However, some mollicutes appear to be in the process of losing their rRNA methyltransferase and, for instance, this is seen as a degenerate nonfunctional version of the *rlmFO* gene in *S. citri* (Figure 4) and what appears to be an *rlmD* pseudogene in some of the *Mycoplasma hyorhinis* strains (Figure 2). No RlmC homolog was identified in any of the mollicutes, nor were there any homologs of the dual-specific enzyme RlmCD seen in *B. subtilis* [36] and *Streptococcus pneumoniae* [57]. Consistent with this, MS analyses of the G725-C772 in domain II of the mollicutes 23S rRNAs confirmed that there was no modification at U747, or at any other nucleotide in this region (not shown). 

Close to the U1939 region of the 23S rRNA, two other nucleotide modifications were identified in some of the mollicutes. The m^3^U1915 modification, which is usually dependent on the prior isomerization of U1915 to [58,59], lies close to Cm1920 within helix 69. Helix 69 is essential for ribosomal subunit interaction, P-site tRNA binding, and recycling of the ribosome after translational termination, and these modifications are thought to facilitate these processes [60,61,62]. Intriguingly, combinations of these modifications in mollicutes ranged from all three (m^3^U1915, Cm1920, and m^5^U1939) in *M. capricolum* subsp. *capricolum* and *A.* *laidlawii*; to various pairs of two (Cm1920/m^5^U1939 or m^3^U1915/m^5^U1939), respectively, in *Me. florum* and *M. auris*; to different single modifications in *S. citri* (Cm1920) and *M. agalactiae* (m^5^U1939); to none at all in *M. gallisepticum* (Figure 4 and Figure S8). This array can be compared to bacteria with larger genomes where *B. subtilis* has all three modifications, whereas *E. coli* and other Enterobacteria make do with m^3^1915 and m^5^U1939 [63]. Surprisingly, a gene encoding the RlmH enzyme responsible for m^3^U1915 modification was predicted in *Me. florum*, *S. citri*, and *M. agalactiae*, while this site was shown to remain unmodified. Multiple attempts of alignment of RlmH proteins, functional domain prediction, and model reconstruction did not show any potential differences in active-site residues that could explain the experimental results. RlmH protein was detected in proteomic studies for *M. capricolum* subsp. *capricolum* (Sirand-Pugnet et al., unpublished results) and *S. citri* (Béven et al., unpublished results) but not for *Me. florum* (Matteau et al., unpublished results). Therefore, the absence of detectable m^3^U1915 modification by RlmH may be due to a low expression level of the protein (in *Me. florum*) or point mutations causing a loss or change of its function (in *S. citri* and *M. agalactiae*). 

### 3.5. Binding Sites of Folate and Flavin are Conserved in TrmFO-like Proteins

**Figure 5:**
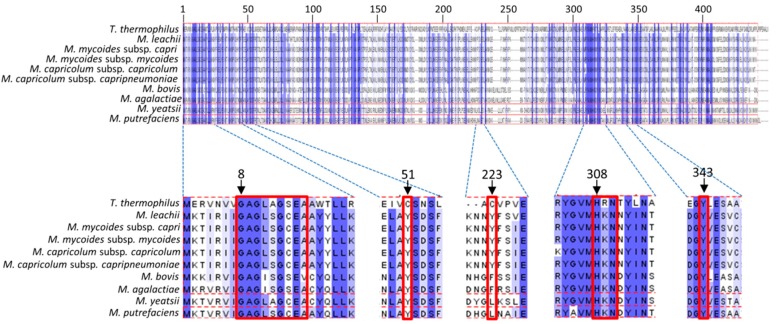
Conserved positions in TrmFO-like homologs. Multiple alignment of TrmFO-like proteins with reference TrmFO from *T. thermophilus* was implemented into Jalview to get an overview of the conserved positions. Amino acids conserved at >50% are indicated in blue. Important regions described in the main text are enlarged below the overview alignment. Positions indicated by arrows correspond to numbering in the reference TrmFO protein of *T. thermophilus.*

The empirical analyses of the mollicutes rRNAs and tRNAs confirmed the functions of the TrmFO and RlmFO enzyme subgroups in targeting, respectively, U1939 in 23S rRNA and U54 in tRNA. However, the TrmFO-like subgroup modifies neither of these sites nor U747, and its function remains unknown. For example, *M. capricolum* subsp. *capricolum* encodes the TrmFO-like protein MCAP_0613 in addition to the RlmFO protein MCAP_0476 that adds the m^5^U1939 rRNA modification, and lacks the m^5^U54-tRNA modification (Figure 3) [37]. Aligning the nine available TrmFO-like sequences from different *Mycoplasma* spp. with the sequence of canonical m^5^U54 flavin- and folate-dependent methyltransferase TrmFO from *Thermus thermophilus* (TrmFO_Tt_) showed that the TrmFO-like proteins are approximately twenty residues shorter than TrmFO_Tt_ (Figure 5). Structural information is available for TrmFO_Tt_ (PDB: 3G5S), and this enzyme exhibits roughly 30% sequence identity with the TrmFO-like proteins. 

We note several key points from the alignment of the TrmFO_Tt_ and TrmFO-like protein sequences (Figure 5). First, the GAGx[A/S]GxE[A/V] motif involved in the recognition of the pyrophosphate group of FAD is conserved in TrmFO-like proteins. Second, the residues H308, R309 or K309 and N310 that are specifically involved in the folate binding are also strictly conserved. Here, the TrmFO_Tt_ crystal structure shows that the side chain of H308 is rotated from its original position in the free-form structure to interact with the pteridine moiety [29]. Additionally, the R309 residue of TrmFO_Tt_ is replaced in the TrmFO-like sequences by lysine, another positively charged residue. Finally, the two catalytic cysteines C51 and C223, which are essential for the U54 methylating activity of TrmFO, are replaced by tyrosine residues in all the TrmFO-like proteins. In TrmFO, C51 acts as a general base and C223 plays the role of a nucleophile that activates the C5-carbon of the uracil target [39,41]. A cysteine residue at position 195-196 of the alignment seems to be conserved in both TrmFO subgroups, but this residue is located far from the active site and is not involved in *B. subtilis* TrmFO catalysis [41]. 

**Figure 6:**
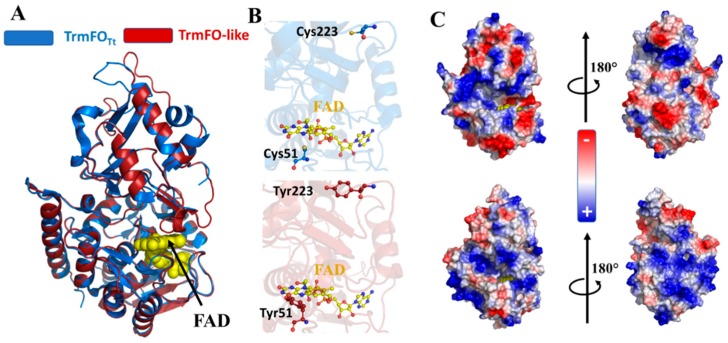
Structural model of TrmFO-like protein. (**A**) Structural overlay of the crystal structure of *T. thermophilus* TrmFO (blue) with the 3D model of *M. capricolum* subsp. *capricolum* TrmFO-like protein MCAP_0613 (red). The FAD coenzyme is represented as ball sticks in yellow. (**B**) Location of the two cysteines (top panel) that are strictly conserved and required for m^5^U54-tRNA methylation activity in TrmFO_Tt_. The lower panel shows the two tyrosine residues at the corresponding locations in TrmFO-like proteins. (**C**) Electrostatic surface of TrmFO_Tt_ (top) and the TrmFO-like protein MCAP_0613 from *M. capricolum* (bottom).

The substitution of C51 and C223 with tyrosines is illustrated in the structural model of the TrmFO-like protein MCAP_0613 (Figure 6) that is based on the TrmFO_Tt_ crystal structure (PDB: 3G5S).

The model was built using the fully automated protein structure homology-modelling server, SWISS-MODEL, achieving acceptable reliability values with GMQE and QMEAN scoring functions of nearly 0.7 and -3.28, respectively. Alignment of the TrmFO-like model against the crystal structure of TrmFO_Tt_ does not reveal any major changes (RMSD = 0.353 Å, over 370 atoms), which would indicate that TrmFO-like proteins adopt the same structural topology seen in the TrmFO/RlmFO subgroups. Compared to TrmFO_Tt_, the identities and spatial locations of all of the residues required to bind FAD and THF are fully conserved in TrmFO-like proteins. Notably, the peculiar Y343 residue that stacks against the isoalloxazine ring in TrmFO_Tt_ and plays an essential role in maintaining active redox state of FAD [64,65,66], is also preserved in TrmFO-like proteins and could feasibly have a similar function. The structural model shows that the two tyrosine residues, Y51 and Y223, occupy positions identical to the two cysteines that they replace in TrmFO_Tt_.

Our sequence and structural analyses suggest that TrmFO-like proteins are similar to TrmFO folate- and FAD-binding proteins, although their enzymatic functions differ. Synteny analysis did not give any clear hint about TrmFO-like function, although the gene immediately downstream of the *trmFO*-like one possibly encodes an EcfS-binding component of a folate ECF transporter (Figure 5), reinforcing the putative connection in folate metabolism. 

### 3.6. Modeling of TrmFO-Like Protein Structures Indicates Diverse Functions

**Figure 7:**
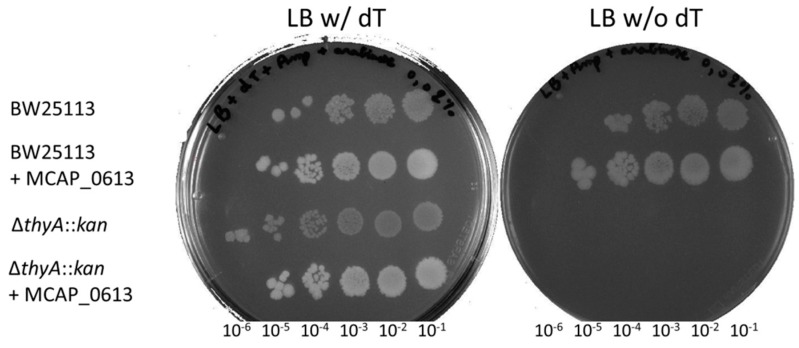
Complementation tests of ∆*thyA*::kan *E. coli* strain. Cells were grown as described in Materials and Methods. Serial dilutions were spotted from right to left, as indicated.

As a prelude to methylation by ThyX, uracil is activated via polarization of two arginine residues within the enzyme’s active site. This differs from that mechanism of ThyA, TrmFO/RlmFO, RlmC, and RlmD, which involves the use of a nucleophile (Table 1). The replacement of both cysteines by two tyrosines (Figure 6) led us to first hypothesize that TrmFO-like proteins could function as 5-methyluracil methyltransferases via a mechanism of uracil activation that differs from ThyX and ThyA (Figure 1). This hypothesis was tested genetically by attempting to suppress the dT auxotrophy of an *E. coli thyA* mutant by expressing the MCAP_0613 gene in *trans*. However, no suppression of thymidine auxotrophy was observed, although expressing MCAP_0613 both in BW25113 and *∆thyA* cells in the presence of dT did lead to higher cell densities both on plates and in liquid cultures density (Figure 7).

The absence of thymidylate synthase activity led us to explore a potential implication of this protein in nucleic acid metabolism. Accordingly, we calculated the electrostatic surface of TrmFO-like models, and this revealed two notable features on the protein from *M. capricolum* subsp. *capricolum* (Figure 6C). First, similar to TrmFO_Tt_, the TrmFO-like protein harbors electropositive patches that surround its active site, suggesting that the TrmFO-like protein could also bind nucleic acids. However, this electropositive surface extends around the TrmFO-like protein and is accessible to the solvent, whereas in TrmFO_Tt_ there is a negatively charged surface on the face opposite to the active site. Taking this idea further, we generated models to analyze the surface electrostatic potentials of TrmFO-like proteins from *M. mycoides*, *M. yeatsii*, *M. putrefaciens*, *M. bovis*, and *M. agalactiae*, obtaining QMQE and QMEAN values that indicate that the models are reliable (Table S4). The TrmFO-like proteins, with the exception of the *M. bovis* and *M. agalactiae* homologs, exhibit similar electrostatic surfaces with positively charged patches around the FAD-binding site **(**Figure S8). Unexpectedly, the TrmFO-like models for *M. bovis* and *M. agalactiae* have electronegative patches formed by a pair of glutamate residues, E204/E283 (Table S1), located in the inserted domain at the distal side of FAD. This would reduce significantly the electropositive surface of these proteins compared to the other TrmFO-like proteins. Consistently, these results could indicate that the *M. bovis* and *M. agalactiae* TrmFO-like proteins have progressively lost tRNA-binding capability while specializing in another cellular function that requires both flavin and folate. Further biochemical and physicochemical studies are required to test this hypothesis.

## 4. Discussion

Taking into account the phylogenetic data, the genomic context, and the experimental validation presented here, we can propose different scenarios for the reductive evolution of the COG2265 and COG1206 families from bacteria with larger genomes to mollicutes. Evolution of the SAM-dependent methyltransferases (COG2265) seems rather simple. We hypothesize that one or more of the bacterial genes *rlmC*, *rlmD*, *rlmCD*, and *YfjO*, but not *trmA*, were present in the ancestor of mollicutes (Figure 2). This is in agreement with the origin of mollicutes from Gram-positive ancestors. The gene coding for YfjO was duplicated several times only during the evolution of the Acholeplasma branch and was completely lost in Phytoplasmas (node 2) as well as in all other mollicutes examined (node 38). The function of the different YfjO-like paralogs of *Acholeplasma* spp. (at least three) is not yet understood and, with the exception of the loss of RNA-binding TRAM domains in the A2 group, no major differences were observed in the residues within the active site of these proteins (Figure S10). It is of course possible that these YfjO-like paralogs have acquired another function and do not methylate an RNA macromolecule. The other ancestral *rlmC/rlmD/rlmCD* genes, which encode rRNA-specific methyltransferases, were all lost in the ancestors of the Spiroplasma (node 36) and Pneumoniae groups (node 12), whereas in the Hominis group, *rlmD* was kept, still encoding the m^5^U1939-specific methyltransferase (node 27). While this is the most parsimonious scenario explaining the data, a novel acquisition event in the ancestor of the Hominis group cannot be formally excluded. A more recent *rlmD* loss was observed for some species, including *M. pulmonis*, and subgroups of species including *M. ovipneumoniae* and *M. hyopneumoniae* (node 19). A recent gene-essentiality study based on transposon mutagenesis in *M. bovis* indicated that *rlmD* (MBOVJF4278_00748) was not necessary [67], reinforcing the dispensability of these rRNA methylases. 

The evolution of the folate-dependent methyltransferases is more complex than the SAM-dependent ones. Again, in agreement with the origin of mollicutes from Gram-positive ancestors, the formation of m^5^U54 formation in mollicutes tRNAs is encoded only by *trmFO* genes and never by *trmA* genes as in Gram-negative bacteria. This *trmFO* gene was probably present in the ancestor of mollicutes (node 38), and in the ancestors of the Acholeplasma/Phytoplasma (AAP, node 3) and Spiroplasma/Hominis/Pneumoniae (SHP, node 37) groups. During the evolution of the AAP group, *trmFO* was maintained in Acholeplasma species but lost in the ancestor of Phytoplasma (node 2). The general lack of any *trmFO*-related gene in the Hominis and Pneumoniae groups indicates a probable loss in the common ancestor of the two subgroups (node 28). By contrast, the distribution of *trmFO*-related genes in the Spiroplasma phylogenetic group (S) suggests a different stepwise evolution. First, there has been a duplication in the subgroup ancestor (node 36) with the evolution of the *rlmFO* paralog to encode a folate-dependent methyltransferase responsible for the 23S rRNA m^5^U1939 modification. This was followed by a second duplication of *trmFO* or *rlmFO* in the ancestor of the mycoplasmas of ruminants (node 34) with the subsequent evolution of *trmFO*-like paralogs. Cases of gene degradation are also visible in the *S. citri trmFO* and *rlmFO* genes, and for *rlmFO* of *M. capricolum* subsp. *capripneumoniae*. In addition, a *trmFO*-like gene from the Hominis subgroup was probably transferred by Horizontal Gene Transfer (HGT) (discussed below) to the ancestor of *M. agalactiae/M. bovis* and, finally, there was a loss of the original *trmFO* in the ancestor of the Mycoides cluster (node 33). HGT between the ruminant mycoplasmas from the Mycoides cluster and the *M. agalactiae/M. bovis* cluster has been described previously [68,69,70].

The complex evolution among the Spiroplasma phylogenetic group (node 36) may have favored the parallel diversification of their methyltransferases. Indeed, appearance of the unique *rlmFO* paralog encoding a folate-dependent 23S rRNA methylase could correspond to an evolutionary relay to maintain the m^5^U1939 modification within the Spiroplasma group as *rlmD* was being lost [37]. From this point of view, enzymatic activity of RlmD and of RlmFO proteins appears to be mutually exclusive, as described for TrmA and TrmFO above. More striking is the occurrence of the *trmFO*-like paralog in the mycoplasmas of ruminants related to the Mycoides cluster, leading to species with up to three *trmFO*-related genes (i.e., *M. putrefaciens* and *M. yeatsii*). In silico analyses suggest that these TrmFO-like proteins may have conserved a folate- and flavin-dependent methylase activity, however their substrate(s) remain unknown (Figure 6 and Figure S9). We have ruled out a potential role in dTMP synthesis (Figure 7). Interestingly, the presence of important patches of positive charges around the active site surface of TrmFO-like structures from *M. capricolum* subsp. *capricolum*, *M. leachi*, *M. mycoides* subsp. *capri*, *M. yeatsii*, and *M. putrefaciens* suggests that the substrate for these proteins could possibly be a nucleic acid. If TrmFO-like proteins also function as methylases, their methylation mechanism must differ from that of TrmFO/RlmFO given that the nucleophilic cysteine has been replaced with a tyrosine (Figure 6). The putative role of this tyrosine as nucleophile, while unusual in nucleic acids enzymology, is not without precedent as several glycosidases utilize a tyrosine that could act as a catalytic nucleophile [71,72]. Experimental validation of this hypothesis will first require the identification of the TrmFO-like substrate and the development of genetic tools to extend our ability to manipulate TrmFO, RlmFO, and TrmFO-like encoding genes in various mollicutes species.

As mentioned above, genome comparisons indicate that an HGT of a *trmFO*-like gene probably took place from the Mycoides cluster to the phylogenetically remote *M. bovis/M. agalactiae* cluster. Among the genes predicted to have been subjected to HGT between these ruminant pathogens, most are related to virulence, metabolism, and mobile elements and none were known to be related to the processing or maintenance of genetic information [68,69,70]. Further analyses on all available complete genomes of *M. agalactiae* (four strains) and *M. bovis* (eleven strains) indicated that a *trmFO*-like gene has been conserved in these species (Figure S11), suggesting that it is biologically significant. It is possible that the transfer and subsequent fixation of *trmFO*-like genes are not due to a role in RNA methylation but rather fulfil a new moonlighting function (see [73] for examples). Our structural homology models indicate that the TrmFO-like proteins of *M. bovis/M. agalactiae* have a smaller electropositive surface than their counterparts in other mollicutes, which would suggest that they may have lost their nucleic acid-binding capacity while preserving a biological activity that depends on both folate and flavin. 

In relation to the possible moonlighting function of certain methyltransferases, the authors of a recent paper [74] have claimed that the TrmFO-like protein (renamed according to our definition here) functions as an adhesin in *M. bovis*. Their conclusion was mainly driven by the demonstration of a fibronectin-binding activity of TrmFO-like using ELISA and direct adhesion assays on embryonic bovine lung (EBL) cells, including inhibition by anti-TrmFO-like polyclonal antibodies. 

In another study, a transposon library of *M. bovis* strain JF-4278 was recently shown to include a *trmFO*-like disrupted mutant whose ability to bind to primary bovine mammary gland epithelial (bMec) cells was reduced, suggesting this gene is a virulence factor in *M. bovis* [67]. 

## 5. Conclusions

In summary, our genetic and structural analyses illustrate evolutionary schemes in which some mollicutes species have kept, while others have lost, the characteristic ancestral Gram-positive C5-uracil methyltransferases. As a consequence of such changes, several new enzyme paralogs have evolved in branches of the mollicutes, where it can be seen that RlmFO and TrmFO-like proteins in most Spiroplasma and YfjO-like proteins in Acholeplasma remain unique to the mycoplasma/mollicutes clade. The drastic genomic reduction at an earlier stage in the evolution of other mollicutes has led to the loss of their capacity to catalyze C5-uracil methylation, similar to findings for other protein factors and enzymes connected with the protein synthesis machinery [47]. 

This study reinforces the concept that the components of the translation machinery evolve as an integrated unit within a given organism, such that the genetic code is accurately and efficiently translated despite nuanced differences in rRNA and tRNA nucleotide modifications that might be seen in a related organism. Thus, the fact that a single modification can be lost in one species does mean it is not of importance in another species. For example, *rlmH* is missing in several groups of mollicutes but has been identified as an essential gene in *Mycoplasma mycoides* subsp. *capri* (closely related to *M. capricolum*) during the construction of the minimal synthetic bacteria JCVI-Syn3.0 (43) (see also in [47]).

Finally, our present studies on COG2265 and COG1206 methyltransferases reveal clear examples of convergence and divergence of enzyme functions within the different mollicutes phyla. These findings in turn beg the question of what selective advantages such changes might confer upon the different species studied here. Answers will undoubtedly be linked with the individual lifestyles of these fast-evolving bacteria, which are often parasitic and narrowly host-specific. Despite significant breakthroughs in the genome engineering of some mycoplasmas using synthetic biology approaches, most mollicutes are still lacking efficient genetic tools. Our current effort to develop such tools for various mollicutes species will open up new ways to decipher the remarkably diverse repertoires of methyltransferases that have been selected during the evolution of those minimal bacteria.

## Figures and Tables

**Table 1:** Different enzyme families and mechanisms of C5-uracil.

Enzymes	Substrates	Coenzymes	Carbon Donor	Reducing Agent	Uracil Activation
TrmA RlmCD	tRNA (U54) rRNA (U747/1939)	none	SAM	none	Cysteine (Michael addition)
TrmFO RlmFO	tRNA (U54) rRNA (U1939)	FAD	CH_2_THF	NAD(P)H	Cysteine (Michael addition)
ThyA	dUMP	none	CH_2_THF	THF	Cysteine (Michael addition)
ThyX PolB	Dump UMP	FAD	CH_2_THF	NAD(P)H	Arginines (Polarization)

## Supplementary Materials

The following are available online at https://www.mdpi.com/2218-273X/10/4/587/s1, Figure S1: Chemical structures of the cofactors and FAD coenzyme employed by the different C5-uracil methyltransferases, Figure S2: Phylogenetic tree of mollicutes, Figure S3: Phylogenetic analysis of TrmFO-related proteins in mollicutes, Figure S4: Genomic context of *rlmFO* homologs, Figure S5: Genomic context of *trmFO*-like homologs, Figure S6: Genomic context of *trmFO* homologs, Figure S7: Phylogenetic analysis of RlmD/RlmCD/YfjO-related proteins in mollicutes, Figure S8: MALDI-MS analyses for m^3^U1915 and Cm1920 methylations, Figure S9: Electrostatic surface of model of TrmFO-like proteins from different mycoplasma, Figure S10: Conserved cysteines in RlmD-related proteins, Figure S11: Multiple alignments of TrmFO-like proteins from *M. bovis* and *M. agalactiae*, Table S1: List of 39 selected mollicutes, Table S2: Homologs of tRNA and rRNA modification enzymes and some other folate- and S-AdoMet-dependent enzymes in mollicutes, Table S3: Supplementary RLMD/RLMCD/YFJO used for phylogenetic analyses, Table S4: GMQE and QMEAN values for TrmFO-like proteins models.

## References

[1] Hori H. (2014). Methylated nucleosides in tRNA and tRNA methyltransferases. Front. Genet..

[2] Motorin Y., Helm M. (2011). RNA nucleotide methylation. Wiley Interdiscip. Rev. RNA.

[3] Myllykallio H., Sournia P., Heliou A., Liebl U. (2018). Unique features and anti-microbial targeting of folate- and flavin-dependent methyltransferases required for accurate maintenance of genetic information. Front. Microbiol..

[4] Juhling F., Morl M., Hartmann R.K., Sprinzl M., Stadler P.F., Putz J. (2009). tRNAdb 2009: Compilation of tRNA sequences and tRNA genes. Nucleic Acids Res..

[5] Felden B., Hanawa K., Atkins J.F., Himeno H., Muto A., Gesteland R.F., McCloskey J.A., Crain P.F. (1998). Presence and location of modified nucleotides in *Escherichia coli* tmRNA: Structural mimicry with tRNA acceptor branches. EMBO J..

[6] Boccaletto P., Machnicka M.A., Purta E., Piatkowski P., Baginski B., Wirecki T.K., de Crécy-Lagard V., Ross R., Limbach P.A., Kotter A. (2018). MODOMICS: A database of RNA modification pathways. 2017 update. Nucleic Acids Res..

[7] Johnson L., Hayashi H., Söll D. (1970). Isolation and properties of a transfer ribonucleic acid deficient in ribothymidine. Biochemistry.

[8] Vani B.R., Ramakrishnan T., Taya Y., Noguchi S., Yamaizumi Z., Nishimura S. (1979). Occurrence of 1-methyladenosine and absence of ribothymidine in transfer ribonucleic acid of *Mycobacterium smegmatis*. J. Bacteriol..

[9] Hsuchen C.C., Dubin D.T. (1980). Methylation patterns of mycoplasma transfer and ribosomal ribonucleic acid. J. Bacteriol..

[10] Piekna-Przybylska D., Decatur W.A., Fournier M.J. (2008). The 3D rRNA modification maps database: With interactive tools for ribosome analysis. Nucleic Acids Res..

[11] Sergeeva O.V., Bogdanov A.A., Sergiev P.V. (2015). What do we know about ribosomal RNA methylation in *Escherichia coli*?. Biochimie.

[12] Motorin Y., Helm M. (2010). tRNA stabilization by modified nucleotides. Biochemistry.

[13] Chawla M., Oliva R., Bujnicki J.M., Cavallo L. (2015). An atlas of RNA base pairs involving modified nucleobases with optimal geometries and accurate energies. Nucleic Acids Res..

[14] Lorenz C., Lunse C.E., Morl M. (2017). tRNA modifications: Impact on structure and thermal adaptation. Biomolecules.

[15] Whipple J.M., Lane E.A., Chernyakov I., D’Silva S., Phizicky E.M. (2011). The yeast rapid tRNA decay pathway primarily monitors the structural integrity of the acceptor and T-stems of mature tRNA. Genes Dev..

[16] Hopper A.K., Huang H.Y. (2015). Quality control pathways for nucleus-encoded eukaryotic tRNA biosynthesis and subcellular trafficking. Mol. Cell. Biol..

[17] Chen W., Li Y., Li J., Wu L., Wang R., Deng Z., Zhou J. (2016). An unusual UMP C-5 methylase in nucleoside antibiotic polyoxin biosynthesis. Protein Cell.

[18] Hamdane D., Grosjean H., Fontecave M. (2016). Flavin-dependent methylation of RNAs: Complex chemistry for a simple modification. J. Mol. Biol..

[19] Hou Y.M., Perona J.J. (2010). Stereochemical mechanisms of tRNA methyltransferases. FEBS Lett..

[20] Kealey J.T., Gu X., Santi D.V. (1994). Enzymatic mechanism of tRNA (m^5^U54)methyltransferase. Biochimie.

[21] Swinehart W.E., Jackman J.E. (2015). Diversity in mechanism and function of tRNA methyltransferases. RNA Biol..

[22] Carreras C.W., Santi D.V. (1995). The catalytic mechanism and structure of thymidylate synthase. Annu. Rev. Biochem..

[23] Myllykallio H., Leduc D., Filee J., Liebl U. (2003). Life without dihydrofolate reductase FolA. Trends Microbiol..

[24] Myllykallio H., Lipowski G., Leduc D., Filee J., Forterre P., Liebl U. (2002). An alternative flavin-dependent mechanism for thymidylate synthesis. Science.

[25] Mishanina T.V., Yu L., Karunaratne K., Mondal D., Corcoran J.M., Choi M.A., Kohen A. (2016). An unprecedented mechanism of nucleotide methylation in organisms containing *thyX*. Science.

[26] Bou-Nader C., Cornu D., Guerineau V., Fogeron T., Fontecave M., Hamdane D. (2017). Enzyme activation with a synthetic catalytic co-enzyme intermediate: Nucleotide methylation by flavoenzymes. Angew. Chem. Int. Ed. Engl..

[27] Conrad J.A., Ortiz-Maldonado M., Hoppe S.W., Palfey B.A. (2014). Detection of intermediates in the oxidative half-reaction of the FAD-dependent thymidylate synthase from *Thermotoga maritima*: Carbon transfer without covalent pyrimidine activation. Biochemistry.

[28] Delk A.S., Nagle D.P., Rabinowitz J.C. (1980). Methylenetetrahydrofolate-dependent biosynthesis of ribothymidine in transfer RNA of *Streptococcus faecalis*. Evidence for reduction of the 1-carbon unit by FADH2. J. Biol. Chem..

[29] Nishimasu H., Ishitani R., Yamashita K., Iwashita C., Hirata A., Hori H., Nureki O. (2009). Atomic structure of a folate/FAD-dependent tRNA T54 methyltransferase. Proc. Natl. Acad. Sci. USA.

[30] Urbonavicius J., Skouloubris S., Myllykallio H., Grosjean H. (2005). Identification of a novel gene encoding a flavin-dependent tRNA:m^5^U methyltransferase in bacteria--evolutionary implications. Nucleic Acids Res..

[31] Björk G.R. (1975). Transductional mapping of gene trmA responsible for the production of 5-methyluridine in transfer ribonucleic acid of *Escherichia coli*. J. Bacteriol..

[32] Ny T., Bjork G.R. (1980). Cloning and restriction mapping of the *trmA* gene coding for transfer ribonucleic acid (5-methyluridine)-methyltransferase in *Escherichia coli* K-12. J. Bacteriol..

[33] Ranaei-Siadat E., Fabret C., Seijo B., Dardel F., Grosjean H., Nonin-Lecomte S. (2013). RNA-methyltransferase TrmA is a dual-specific enzyme responsible for C5-methylation of uridine in both tmRNA and tRNA. RNA Biol..

[34] Agarwalla S., Kealey J.T., Santi D.V., Stroud R.M. (2002). Characterization of the 23 S ribosomal RNA m5U1939 methyltransferase from *Escherichia coli*. J. Biol. Chem..

[35] Madsen C.T., Mengel-Jorgensen J., Kirpekar F., Douthwaite S. (2003). Identifying the methyltransferases for m(5)U747 and m(5)U1939 in 23S rRNA using MALDI mass spectrometry. Nucleic Acids Res..

[36] Desmolaize B., Fabret C., Bregeon D., Rose S., Grosjean H., Douthwaite S. (2011). A single methyltransferase YefA (RlmCD) catalyses both m^5^U747 and m^5^U1939 modifications in *Bacillus subtilis* 23S rRNA. Nucleic Acids Res..

[37] Lartigue C., Lebaudy A., Blanchard A., El Yacoubi B., Rose S., Grosjean H., Douthwaite S. (2014). The flavoprotein Mcap0476 (RlmFO) catalyzes m^5^U1939 modification in *Mycoplasma capricolum* 23S rRNA. Nucleic Acids Res..

[38] Urbonavicius J., Brochier-Armanet C., Skouloubris S., Myllykallio H., Grosjean H. (2007). In vitro detection of the enzymatic activity of folate-dependent tRNA (Uracil-54,-C5)-methyltransferase: Evolutionary implications. Methods Enzymol..

[39] Hamdane D., Argentini M., Cornu D., Golinelli-Pimpaneau B., Fontecave M. (2012). FAD/folate-dependent tRNA methyltransferase: Flavin as a new methyl-transfer agent. J. Am. Chem. Soc..

[40] Hamdane D., Bruch E., Un S., Field M., Fontecave M. (2013). Activation of a unique flavin-dependent tRNA-methylating agent. Biochemistry.

[41] Hamdane D., Argentini M., Cornu D., Myllykallio H., Skouloubris S., Hui-Bon-Hoa G., Golinelli-Pimpaneau B. (2011). Insights into folate/FAD-dependent tRNA methyltransferase mechanism: Role of two highly conserved cysteines in catalysis. J. Biol. Chem..

[42] Kamminga T., Koehorst J.J., Vermeij P., Slagman S.J., Martins Dos Santos V.A., Bijlsma J.J., Schaap P.J. (2017). Persistence of functional protein domains in Mycoplasma species and their role in host specificity and synthetic minimal life. Front. Cell. Infect. Microbiol..

[43] Hutchison C.A., Chuang R.Y., Noskov V.N., Assad-Garcia N., Deerinck T.J., Ellisman M.H., Gill J., Kannan K., Karas B.J., Ma L. (2016). Design and synthesis of a minimal bacterial genome. Science.

[44] Chen L.L., Chung W.C., Lin C.P., Kuo C.H. (2012). Comparative analysis of gene content evolution in phytoplasmas and mycoplasmas. PLoS ONE.

[45] Barre A., de Daruvar A., Blanchard A. (2004). MolliGen, a database dedicated to the comparative genomics of Mollicutes. Nucleic Acids Res..

[46] Uchiyama I., Mihara M., Nishide H., Chiba H. (2015). MBGD update 2015: Microbial genome database for flexible ortholog analysis utilizing a diverse set of genomic data. Nucleic Acids Res..

[47] Grosjean H., Breton M., Sirand-Pugnet P., Tardy F., Thiaucourt F., Citti C., Barre A., Yoshizawa S., Fourmy D., de Crecy-Lagard V. (2014). Predicting the minimal translation apparatus: Lessons from the reductive evolution of mollicutes. PLoS Genet..

[48] Castresana J. (2000). Selection of conserved blocks from multiple alignments for their use in phylogenetic analysis. Mol. Biol. Evol..

[49] Dereeper A., Guignon V., Blanc G., Audic S., Buffet S., Chevenet F., Dufayard J.F., Guindon S., Lefort V., Lescot M. (2008). Phylogeny.fr: Robust phylogenetic analysis for the non-specialist. Nucleic Acids Res..

[50] Waterhouse A.M., Procter J.B., Martin D.M., Clamp M., Barton G.J. (2009). Jalview Version 2—A multiple sequence alignment editor and analysis workbench. Bioinformatics.

[51] Bienert S., Waterhouse A., de Beer T.A., Tauriello G., Studer G., Bordoli L., Schwede T. (2017). The SWISS-MODEL Repository-new features and functionality. Nucleic Acids Res..

[52] Baker N.A., Sept D., Joseph S., Holst M.J., McCammon J.A. (2001). Electrostatics of nanosystems: Application to microtubules and the ribosome. Proc. Natl. Acad. Sci. USA.

[53] Giessing A.M., Jensen S.S., Rasmussen A., Hansen L.H., Gondela A., Long K., Vester B., Kirpekar F. (2009). Identification of 8-methyladenosine as the modification catalyzed by the radical SAM methyltransferase Cfr that confers antibiotic resistance in bacteria. RNA.

[54] Andersen T.E., Porse B.T., Kirpekar F. (2004). A novel partial modification at C2501 in *Escherichia coli* 23S ribosomal RNA. RNA.

[55] Douthwaite S., Kirpekar F. (2007). Identifying modifications in RNA by MALDI mass spectrometry. Methods Enzymol..

[56] Kirpekar F., Douthwaite S., Roepstorff P. (2000). Mapping posttranscriptional modifications in 5S ribosomal RNA by MALDI mass spectrometry. RNA.

[57] Shoji T., Takaya A., Sato Y., Kimura S., Suzuki T., Yamamoto T. (2015). RlmCD-mediated U747 methylation promotes efficient G748 methylation by methyltransferase RlmAII in 23S rRNA in *Streptococcus pneumoniae*; interplay between two rRNA methylations responsible for telithromycin susceptibility. Nucleic Acids Res..

[58] Ero R., Peil L., Liiv A., Remme J. (2008). Identification of pseudouridine methyltransferase in *Escherichia coli*. RNA.

[59] Purta E., Kaminska K.H., Kasprzak J.M., Bujnicki J.M., Douthwaite S. (2008). YbeA is the m^3^Psi methyltransferase RlmH that targets nucleotide 1915 in 23S rRNA. RNA.

[60] Ali I.K., Lancaster L., Feinberg J., Joseph S., Noller H.F. (2006). Deletion of a conserved, central ribosomal intersubunit RNA bridge. Mol. Cell.

[61] Ero R., Leppik M., Liiv A., Remme J. (2010). Specificity and kinetics of 23S rRNA modification enzymes RlmH and RluD. RNA.

[62] Liu Q., Fredrick K. (2016). Intersubunit bridges of the bacterial ribosome. J. Mol. Biol..

[63] Purta E., O’Connor M., Bujnicki J.M., Douthwaite S. (2009). YgdE is the 2′-O-ribose methyltransferase RlmM specific for nucleotide C2498 in bacterial 23S rRNA. Mol. Microbiol..

[64] Hamdane D., Bou-Nader C., Cornu D., Hui-Bon-Hoa G., Fontecave M. (2015). Flavin-Protein Complexes: Aromatic stacking assisted by a hydrogen bond. Biochemistry.

[65] Nag L., Sournia P., Myllykallio H., Liebl U., Vos M.H. (2017). Identification of the TyrOH(*+) Radical Cation in the Flavoenzyme TrmFO. J. Am. Chem. Soc..

[66] Dozova N., Lacombat F., Bou-Nader C., Hamdane D., Plaza P. (2019). Ultrafast photoinduced flavin dynamics in the unusual active site of the tRNA methyltransferase TrmFO. Phys. Chem. Chem. Phys. PCCP.

[67] Josi C., Burki S., Vidal S., Dordet-Frisoni E., Citti C., Falquet L., Pilo P. (2019). Large-scale analysis of the *Mycoplasma bovis* genome identified non-essential, adhesion- and virulence-related genes. Front. Microbiol..

[68] Thomas A., Linden A., Mainil J., Bischof D.F., Frey J., Vilei E.M. (2005). *Mycoplasma bovis* shares insertion sequences with *Mycoplasma agalactiae* and *Mycoplasma mycoides* subsp. mycoides SC: Evolutionary and developmental aspects. FEMS Microbiol. Lett..

[69] Sirand-Pugnet P., Lartigue C., Marenda M., Jacob D., Barre A., Barbe V., Schenowitz C., Mangenot S., Couloux A., Segurens B. (2007). Being pathogenic, plastic, and sexual while living with a nearly minimal bacterial genome. PLoS Genet..

[70] Lo W.S., Gasparich G.E., Kuo C.H. (2018). Convergent evolution among ruminant-pathogenic Mycoplasma involved extensive gene content changes. Genome Biol. Evol..

[71] Watts A.G., Damager I., Amaya M.L., Buschiazzo A., Alzari P., Frasch A.C., Withers S.G. (2003). *Trypanosoma cruzi* trans-sialidase operates through a covalent sialyl-enzyme intermediate: Tyrosine is the catalytic nucleophile. J. Am. Chem. Soc..

[72] Amaya M.F., Watts A.G., Damager I., Wehenkel A., Nguyen T., Buschiazzo A., Paris G., Frasch A.C., Withers S.G., Alzari P.M. (2004). Structural insights into the catalytic mechanism of *Trypanosoma cruzi* trans-sialidase. Structure.

[73] Koliadenko V., Wilanowski T. (2020). Additional functions of selected proteins involved in DNA repair. Free Radic. Biol. Med..

[74] Guo Y., Zhu H., Wang J., Huang J., Khan F.A., Zhang J., Guo A., Chen X. (2017). TrmFO, a fibronectin-binding adhesin of *Mycoplasma bovis*. Int. J. Mol. Sci..

