# Supplementary material for: Reductive Evolution and Diversification of C5-Uracil Methylation in the Nucleic Acids of Mollicutes
Source: Biomolecules. 2020 Apr 10;10(4):587. doi: 10.3390/biom10040587 (PMC7226160; doi:10.3390/biom10040587)
Supplement: Supplementary file 1 [file biomolecules-10-00587-s001.zip › FIG_SUP_revision/Fig S7 Phylo Tree RLMD v4.pdf]

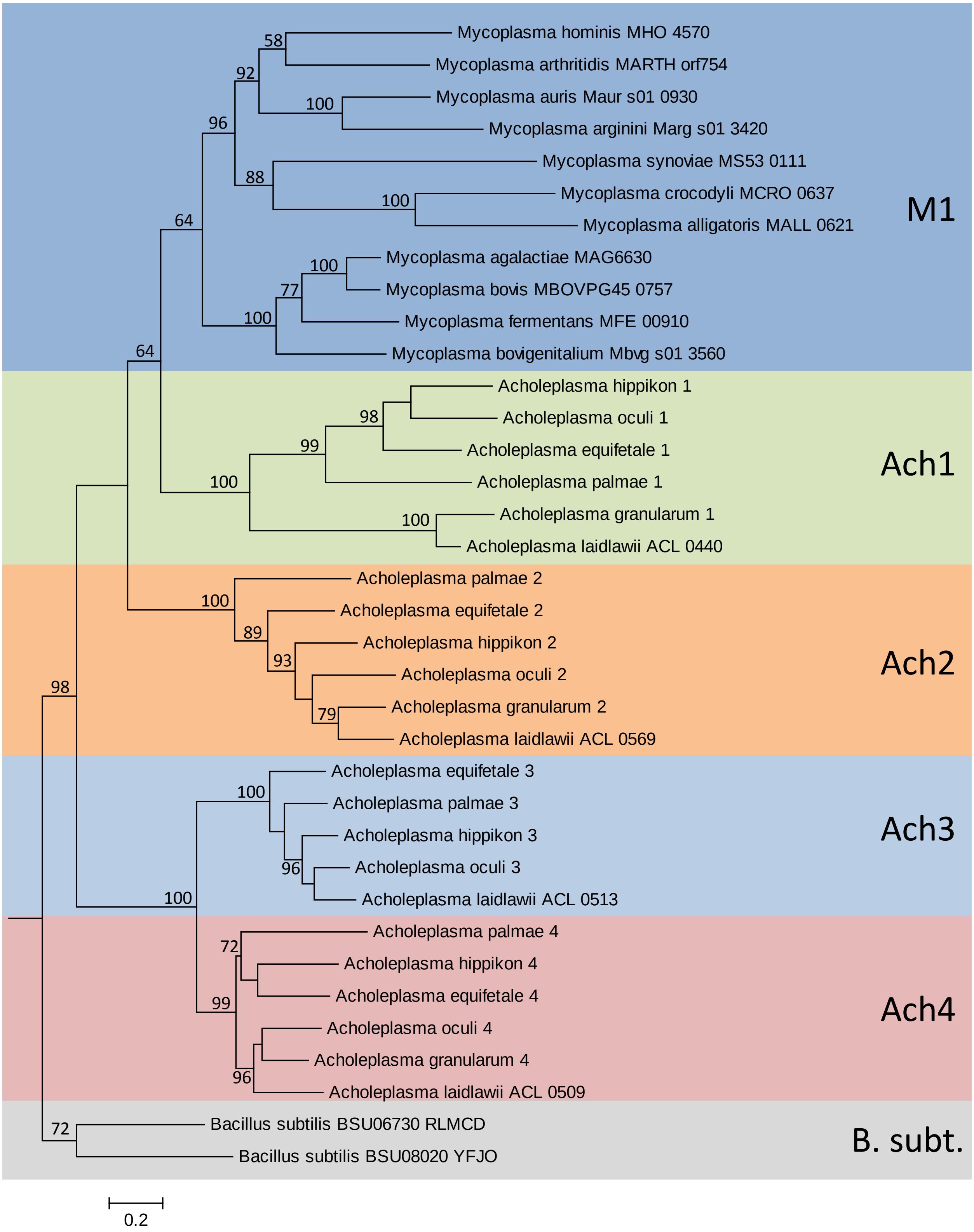

Figure S7. Phylogenetic analysis of RlmD/RlmCD/YfjO- related CDS in mollicutes. Alignment was obtained with MUSCLE and phylogenetic tree was inferred using the maximum likelihood method using the PhyML programme implemented in phylogeny.fr. Statistical aLRT values >50% are indicated on nodes. Main groups of homologs identified are indicated.
