# Supplementary material for: Reductive Evolution and Diversification of C5-Uracil Methylation in the Nucleic Acids of Mollicutes
Source: Biomolecules. 2020 Apr 10;10(4):587. doi: 10.3390/biom10040587 (PMC7226160; doi:10.3390/biom10040587)
Supplement: Supplementary file 1 [file biomolecules-10-00587-s001.zip › FIG_SUP_revision/Fig S2 Phylogenetic tree Mollicutes v1.pdf]

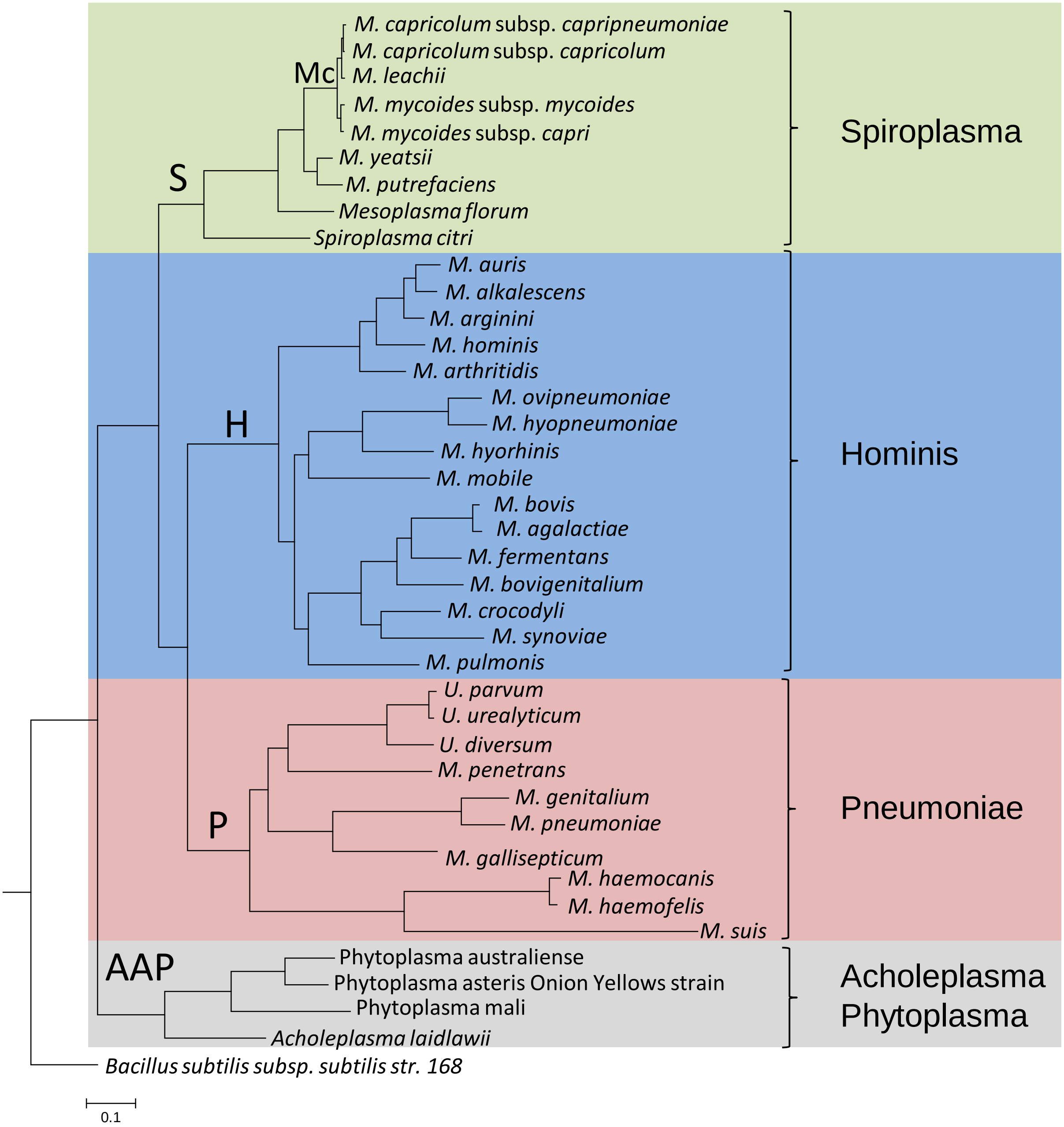

Figure S2. Phylogenetic tree of mollicutes. The phylogenetic tree was generated using the maximum likelihood method from the concatenated multiple sequence alignments of selected 79 orthologous protein involved in translation as described in Grosjean et al. 2014. Main phylogenetic groups are indicated, S, Spiroplasma, H, Hominis, P, Pneumoniae, AAP, Acholeplasma/Phytoplasma. The so-called Mycoides cluster (Mc) includes 5 ruminant mycoplasmas. *B. subtilis* was used as an outgroup.
