# Supplementary material for: Reductive Evolution and Diversification of C5-Uracil Methylation in the Nucleic Acids of Mollicutes
Source: Biomolecules. 2020 Apr 10;10(4):587. doi: 10.3390/biom10040587 (PMC7226160; doi:10.3390/biom10040587)
Supplement: Supplementary file 1 [file biomolecules-10-00587-s001.zip › FIG_SUP_revision/Fig S10 Alignment RlmD YfJO-like v3.pdf]

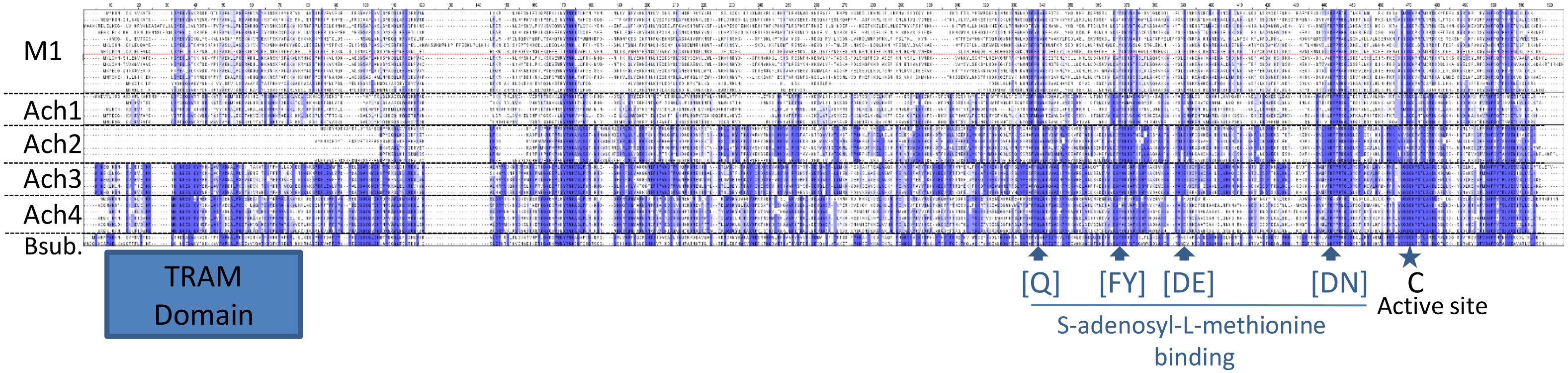


**Figure S10**. Conserved cysteines in RlmD-related proteins. Multiple alignment used for the construction of the phylogenetic tree presented Figure 3 was implemented into Jalview to get an overview of the conserved positions. Amino-acids conserved at >50% are indicated in blue. Functionnal domain TRAM and important amino-acids are indicated underneath the alignment.
