# Supplementary material for: Reductive Evolution and Diversification of C5-Uracil Methylation in the Nucleic Acids of Mollicutes
Source: Biomolecules. 2020 Apr 10;10(4):587. doi: 10.3390/biom10040587 (PMC7226160; doi:10.3390/biom10040587)
Supplement: Supplementary file 1 [file biomolecules-10-00587-s001.zip › FIG_SUP_revision/Table S4.docx]

**Table S4. GMQE and QMEAN values for TrmFO-like proteins models generated by Swiss-model using PDB:3G5S as a template.**

| **Organism** | **Identity with *Tt*TrmFO (%)** | **GMQE** | **QMEAN** |
| --- | --- | --- | --- |
| *M. capricolum* | 29.4 | 0.68 | -3.22 |
| *M. mycoides* | 30.7 | 0.68 | -3.35 |
| *M. yeatsii* | 31.6 | 0.69 | -3.09 |
| *M. putrefaciens* | 31.7 | 0.68 | -2.92 |
| *M. bovis* | 30.6 | 0.69 | -3.22 |
| *M. agalactiae* | 32.3 | 0.69 | -3.55 |
