# Supplementary material for: Reductive Evolution and Diversification of C5-Uracil Methylation in the Nucleic Acids of Mollicutes
Source: Biomolecules. 2020 Apr 10;10(4):587. doi: 10.3390/biom10040587 (PMC7226160; doi:10.3390/biom10040587)
Supplement: Supplementary file 1 [file biomolecules-10-00587-s001.zip › FIG_SUP_revision/Fig S8 MS U1915 and C1920.pdf]

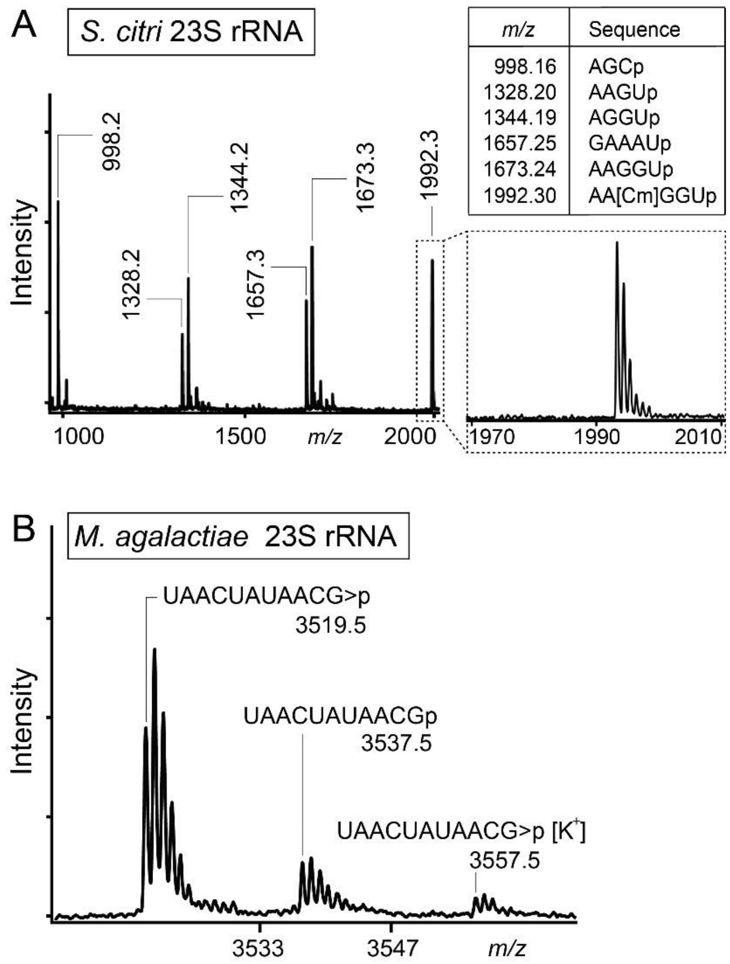

Figure S8 : MALDI-MS analyses for m^{3}U1915 and Cm1920 methylations. (**A**) Spectrum of RNase A fragments from this region (Fig. 4) of the *S. citri* 23S rRNA. The fragment at *m/z* 1992 corresponds to nucleotides 1918-1923 with the sequence UAA[Cm]GGp, where 2´-*O*-methylation at C1920 renders the fragment resistant to RNase A cleavage. In the *M. agalactiae* rRNA, where there is no C1920 methylation, this sequence is cleaved to form UAAp and CGGp that run in the trinucleotide region of the spectrum (not shown). (**B**) RNase T1 digestion gives an additional analysis of this region as shown here in the expanded view of the *M. agalactiae m/z* 3519 peak from Fig. 4. This sequence of nucleotides 1911-1921 contains both U1915 and C1920, and the cyclic 3´-phosphate (>p) fragment at *m/z* 3519 has no modification. Minor peaks are evident corresponding to the same fragment with a linear 3´-phosphate (+ 18 Da) and with an adducted potassium ion (+ 38 Da), but there is no peak corresponding to modification with one or two methyl groups (theoretical *m/z* 3533 and 3547, respectively).
