# Supplementary material for: Reductive Evolution and Diversification of C5-Uracil Methylation in the Nucleic Acids of Mollicutes
Source: Biomolecules. 2020 Apr 10;10(4):587. doi: 10.3390/biom10040587 (PMC7226160; doi:10.3390/biom10040587)
Supplement: Supplementary file 1 [file biomolecules-10-00587-s001.zip › FIG_SUP_revision/Fig S1_ reaction mechanisms.docx]

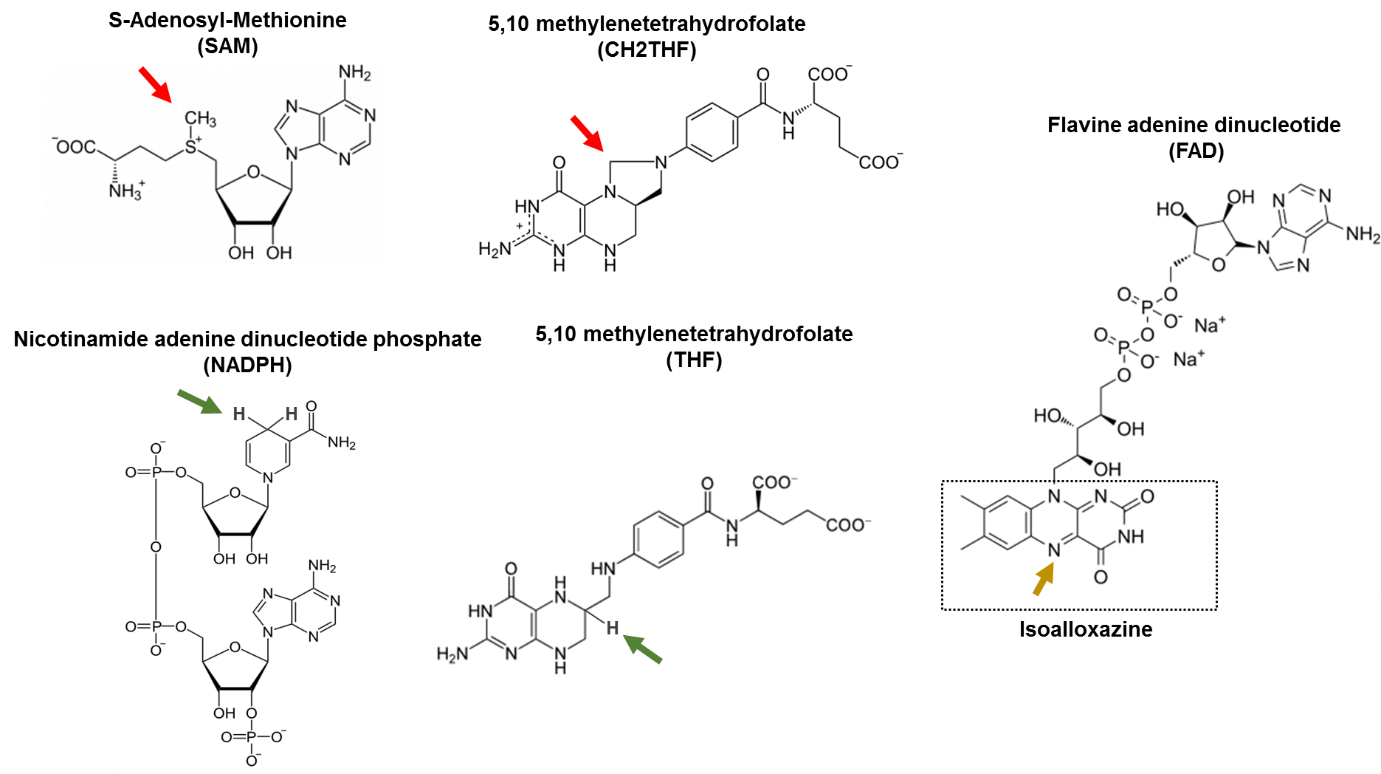


**A**

**B**


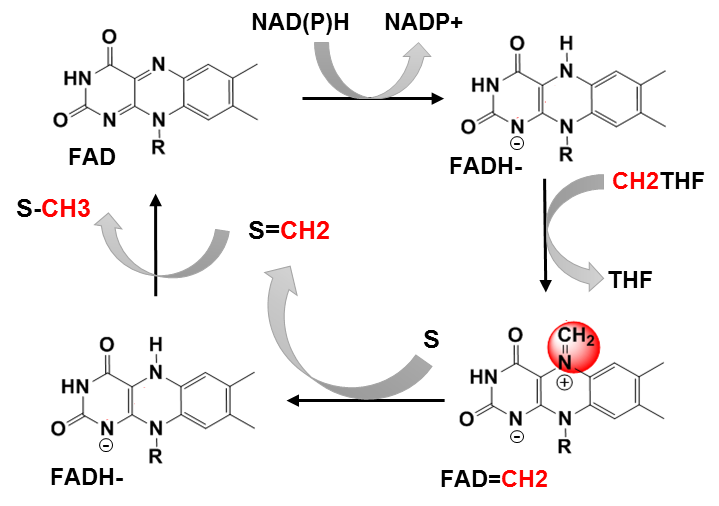


**Figure S1. Chemical structures of the cofactors and FAD coenzyme employed by the different C5-uracil methyltransferases**. (A) Chemical structures of the carbon donor S-adenosylmethionine (SAM) used by TrmA and RlmCD and the 5,10-methylenetetrahydrofolate (CH2THF) used by TrmFO, RlmFO, ThyA, ThyX and PolB. The red arrow indicates the carbon to be donated. Chemical structures of the reducing agent required for the reductive methylations catalyzed by TrmFO, RlmFO, ThyA, ThyX and PolB. The green arrow shows the hydride to be transferred. TrmFO, RlmFO, ThyX and PolB employed NAD(P)H while ThyA used the THF as a source of hydride to reduce the exocyclic methylene group present on the C5-uracil to a methyl group. On the chemical structure of the FAD, the yellow arrow shows the redox active N5 atom involved in the transfer of both hydride and methylene. (B) Chemical structure of the flavin coenzyme species used by TrmFO, RlmFO, ThyX and PolB for the reductive methylation of their respective substrate. The N5 of FAD isoalloxazine ring receives a hydride from NAD(P)H leading to the anionic FADH-, which then accepts the CH2 from CH2THF leading to the flavin iminium FAD=CH2. The latter transfers its electrophilic CH2 to the activated C5-uracil leading to an exocyclic methylene and the recovery of FADH^-^. The methylation ends by the reduction of this exocyclic methylene by a hydride transfer from FADH-.
