# Supplementary material for: Reductive Evolution and Diversification of C5-Uracil Methylation in the Nucleic Acids of Mollicutes
Source: Biomolecules. 2020 Apr 10;10(4):587. doi: 10.3390/biom10040587 (PMC7226160; doi:10.3390/biom10040587)
Supplement: Supplementary file 1 [file biomolecules-10-00587-s001.zip › FIG_SUP_revision/Fig S3 Phylogenetic Tree TrmFO v6.pdf]

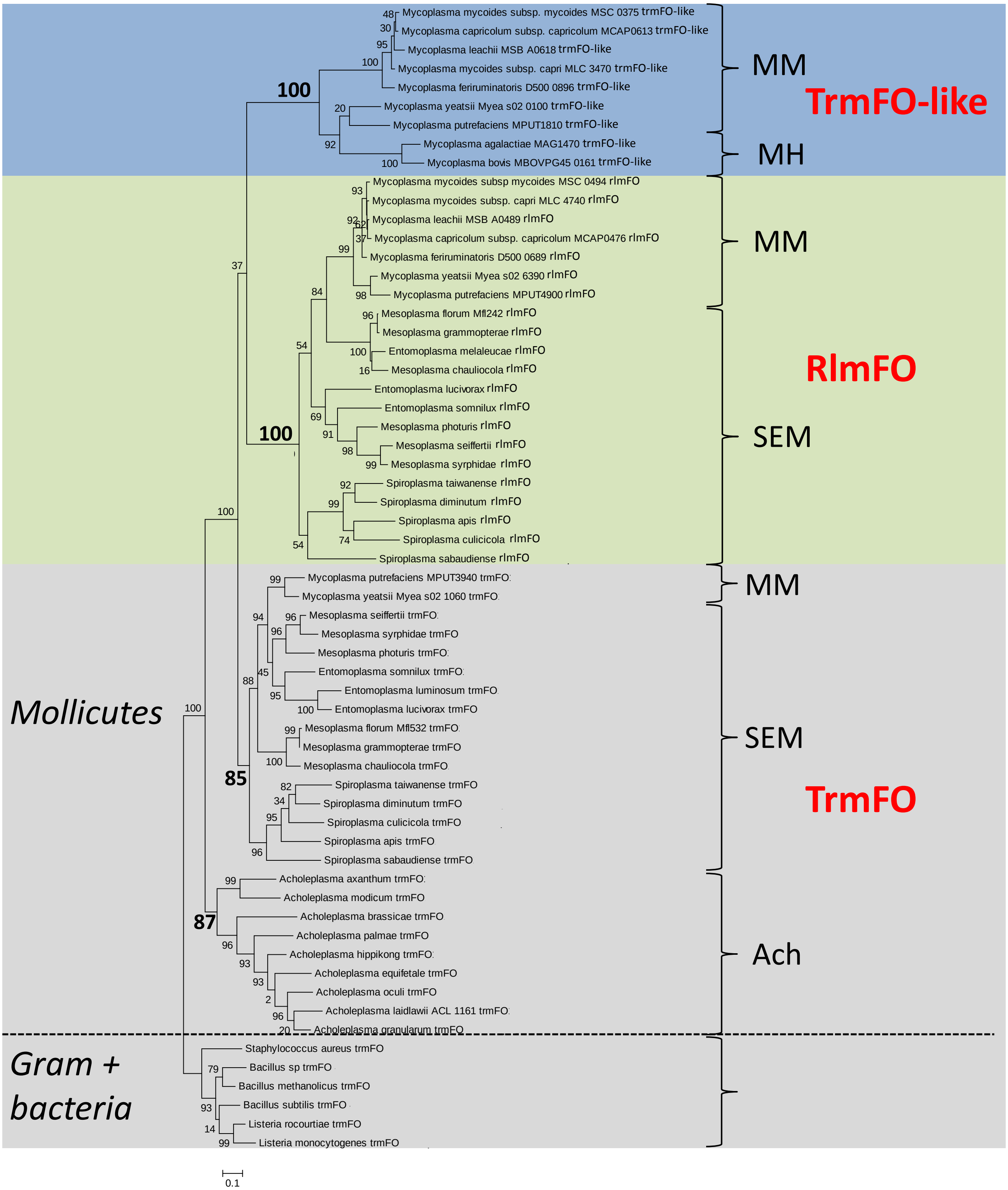

Figure S3. Phylogenetic analysis of TrmFO-related proteins in mollicutes. Alignment was obtained with MUSCLE and cured from unreliable positions using Gblocks; 331 positions were retained. The phylogenetic tree was inferred using the maximum likelihood method using the PhyML programme implemented in phylogeny.fr. Statistical aLRT values are indicated on nodes. Main phylogenetic groups of mollicutes are indicated ; Ach, Acholeplasma ; SEM, Spiroplasma-Entomoplasma-Mesoplasma ; MM, Mycoplasma mycoides-related species ; MH, Mycoplasma hominis-related species. *B. subtilis* TrmFO-related proteins in mollicutes were distributed into three groups of homologs from phylogenetic and synteny analyses; grey, TrmFO; green, RlmFO and blue, TrmFO-like. Mnemonics are indicated for the reference set.
