# Supplementary material for: Reductive Evolution and Diversification of C5-Uracil Methylation in the Nucleic Acids of Mollicutes
Source: Biomolecules. 2020 Apr 10;10(4):587. doi: 10.3390/biom10040587 (PMC7226160; doi:10.3390/biom10040587)
Supplement: Supplementary file 1 [file biomolecules-10-00587-s001.zip › FIG_SUP_revision/Fig S6 Synteny trmFO.pdf]

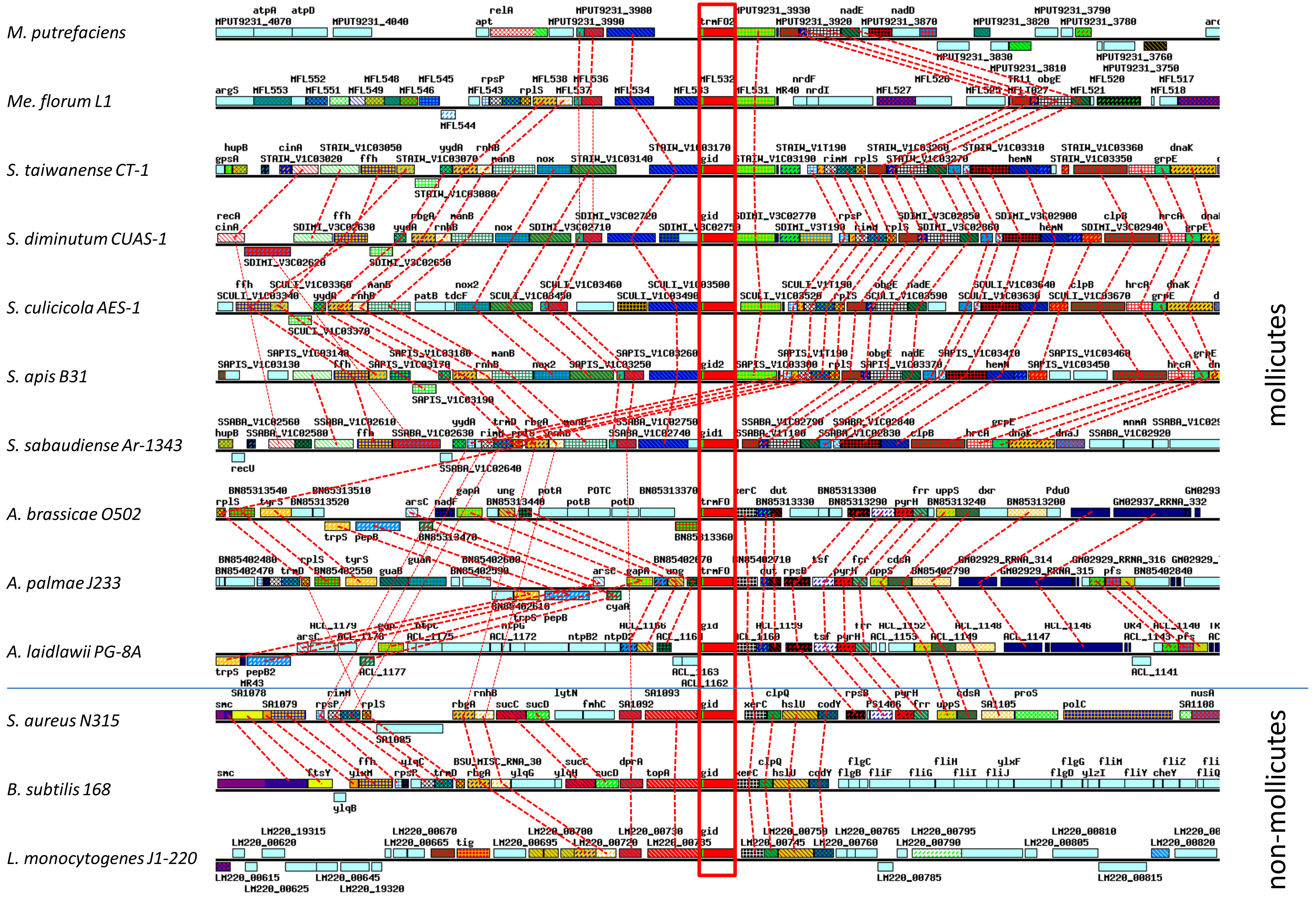

Figure S6. Genomic context of *trmFO* homologs. Analysis of synteny and genome context was performed using the MBGD database. Homologous *trmFO* genes are framed and coloured in red. Other homologs are coloured the same and connected with red dotted lines.
