# Supplementary figures and images for: Reductive Evolution and Diversification of C5-Uracil Methylation in the Nucleic Acids of Mollicutes
Source: Biomolecules. 2020 Apr 10;10(4):587. doi: 10.3390/biom10040587 (PMC7226160; doi:10.3390/biom10040587)

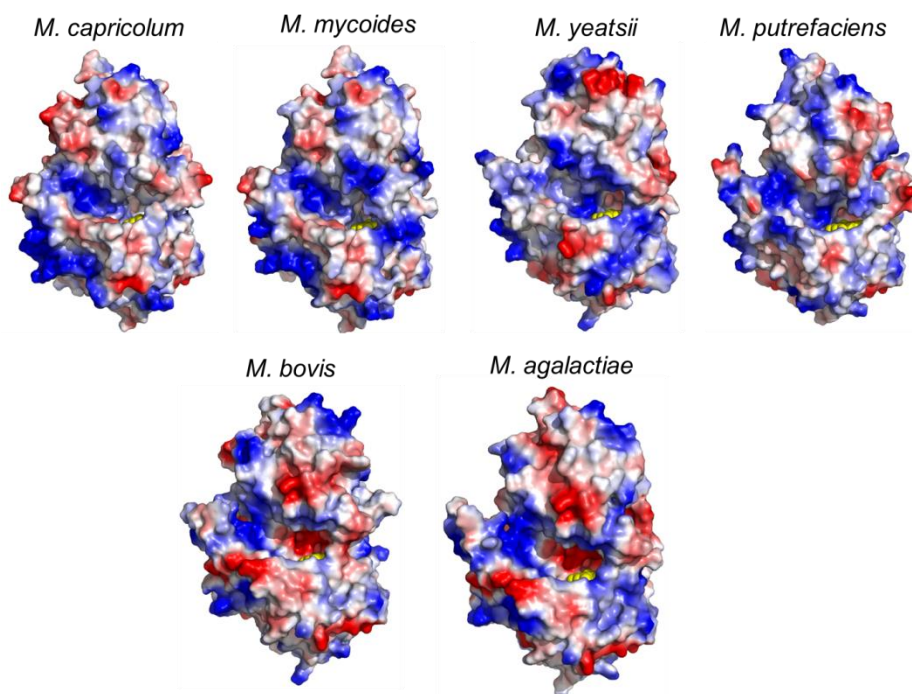

**Figure S9.** Electrostatic surface of model of TrmFO-like proteins from different mycoplasma.

Supplement: Supplementary file 1 [file biomolecules-10-00587-s001.zip › FIG_SUP_revision/Fig S9 protein structures.pdf]
